# An Electrospun Core–Sheath/Janus-Integrated Hierarchical Structure for Asynchronous Mucosal Drug Delivery

**DOI:** 10.3390/pharmaceutics18101333

**Published:** 2026-10-20

**Authors:** Qilin Wang, Tingyu Chen, Ran Dong, Deng-Guang Yu, Sim-Wan Annie Bligh

**Affiliations:** 1 School of Health Science and Engineering, University of Shanghai for Science and Technology, Shanghai 200093, China; 243403174@st.usst.edu.cn (Q.W.); 243403094@st.usst.edu.cn (T.C.); 251620330@st.usst.edu.cn (R.D.); 2 School of Health Sciences, Saint Francis University, Hong Kong 999077, China

**Keywords:** trifluid electrospinning, hierarchic nanostructures, dual-drug compartmentalized loading, asynchronous controlled release, oral mucosal drug delivery, motion sickness

## Abstract

**Background**: It is highly desirable to address the dual therapeutic needs of quickly alleviating clinical symptoms and providing long-lasting effects for motion sickness, as well as to overcome limitations of traditional electrospinning methods—such as easy drug mixing, poor zoning accuracy, and inadequate controlled-release capabilities. **Methods**: This study developed a self-developed trifluid electrospinning device. This device was used to create a dual-drug loaded nanofiber membrane containing diphenhydramine (DP) and scopolamine hydrobromide (SH), featuring a zoned structure composed of a polyvinylpyrrolidone (PVP) crescent side chamber, a round polylactic acid (PLA) sheath chamber, and a round PLA core chamber. **Results**: The results demonstrated that the fibers maintained an intact structure with distinct interfaces, allowing precise zoned loading of the two drugs, which were present in an amorphous dispersed state, indicating good compatibility and structural stability. The nanofiber membrane showed moderate mechanical strength and excellent hydrophilicity, making it suitable for oral mucosal drug delivery. Asynchronous drug delivery, featuring a rapid release of DP (53% at 1 h) and a sustained release of SH (85% at 12 h with no noticeable burst phenomenon), was demonstrated using two types of hierarchically structured nanofibers, each loaded with a single drug to facilitate measurement. The release mechanisms were disclosed for DP and SH from the tri-chamber hierarchical nanofibers. Cell studies confirmed its good biocompatibility. **Conclusions**: This work offers new insights and experimental evidence for developing an effective synergistic oral delivery system for motion sickness and advancing multifunctional nanocarrier drug delivery platforms.

## 1. Introduction

Coaxial electrospinning is a well-established method for creating core–sheath nanofibers and has been extensively used in producing drug-loaded nanofibrous membranes [1,2,3,4,5,6,7]. However, this approach can only form a single core–sheath binary spatial structure and cannot independently separate and load two different therapeutic drugs, which limits its ability to achieve differentiated drug distribution designs. In contract, side-by-side electrospinning has been explored for preparing Janus nanostructures, with two different drugs having their own polymeric matrices for a tailored controlled release profile [8,9,10,11,12,13]. However, due to the diffusion and degradation properties inherent to a single polymer-based monolithic phase, the capacity to control drug release is quite restricted, leading to a relatively short sustained-release duration and an inability to simultaneously fulfill the dual therapeutic needs of rapid drug action and prolonged effectiveness. Thus, a tri-chamber hierarchic nanostructure, an integration of a core–sheath structure and Janus fiber, may combine their advantages to provide an asynchronous delivery of double the active ingredients, with each having their elaborate controlled release profiles for a joint therapeutic effect.

Electrospinning, electrospraying and e-jetting printing comprise the most common three electrohydrodynamic atomization methods that take advantage of the facile interactions between the high voltage electrostatic energy with the working fluids [14,15,16,17,18,19]. There were three initial reasons for the popularity of electrospinning, i.e., its development as part of the nano era, the easy and low-cost setup of electrospinning apparatus, and the unique properties of electrospun nanofibrous mats, such as their small diameter, large porosity, and facility for after-treatment or further conversion [20,21,22,23,24]. Later, multiple-fluid electrospinning became more popular in scientific investigations than the common single-fluid blended processes because of two factors. One is that complex nanostructures and nano devices represent one of the most important directions of nano science and nano techniques [25,26,27]. Secondly, enthusiasm about traditional single-fluid blended processes has moved toward large-scale productions and potential commercial products. As a result, the exploration of multiple fluid electrospinning for creating complex nanostructures is likely to be a hot research topic for the coming several decades.

The current conventional trifluid electrospinning techniques are mostly confined to trifluid parallel or triaxial structural designs [28,29,30,31]. During the spinning and formation process, these structures often face problems such as disturbances at the interfaces between multiple fluids, interpenetration of components, and diffusion and mixing between various drug layers. These issues significantly compromise the uniformity and stability of the multi-chamber nanostructures. More importantly, these sole tri-chamber nanostructures from trifluid electrospinning lack a customizable hierarchical interfacial design, preventing the regulation of drug diffusion pathways through optimizations of polymer formulations and rational allocation of spaces for each chamber. As a result, it is challenging to tailor the starting release time points and the reasonable release time periods of co-loaded drugs, which can exert great influences on the full achievement of synergistic therapeutic effects.

Provoked by this issue, here a tri-chamber hierarchic nanostructure integrated from a core–sheath structure and Janus fiber is developed, and a new trifluid electrospinning method was developed for simple and straightforward fabrication. The spinneret is the most important element in an electrospinning apparatus and benefits from a large number of innovations [32,33]. Its nozzle structures are exploited as the macro templates for duplicating complex nanostructures, such as core–sheath nanofibers from a spinneret with a concentric nozzle [34,35,36,37,38,39], and Janus nanofibers from an eccentric nozzle [40]. To effectively address inherent issues in traditional coaxial electrospinning such as interface fusion, spontaneous drug diffusion across layers, and mutual interference of components, the developed spinneret features a unique design with three independent fluid channels, enabling precise spatial compartmentalization and loading of two functional drugs from the working fluids. Meanwhile, the elaborate hierarchical designs of the nanofibers from the spinneret’s nozzles, through rational matching of hydrophilic/hydrophobic polymer matrices and regulation of hierarchical interface properties, precisely control drug release, diffusion, and matrix degradation behaviors, enabling the capability of tailoring drug controlled-release profiles. We hypothesized that a hierarchical nanostructure integrated from core–sheath and Janus structures could be directly fabricated using a new method of trifluid electrospinning, which was optimized to enhance the temporal synergy of dual-drug asynchronous delivery with their separative “rapid release and long-lasting sustained release”, making the drug delivery system better suited to the clinical treatment needs of motion sickness for rapid symptom relief and long-term efficacy maintenance.

To address the therapeutic need for motion sickness, controlled release combining fast-acting diphenhydramine (DP) with long-lasting scopolamine hydrobromide (SH) was anticipated to be achieved through spatial separation and the rational structural design of tri-chamber architectures. A trifluid electrospinning apparatus was therefore established based on a patented spinneret [41]. Polyvinylpyrrolidone (PVP) and polylactic acid (PLA) were selected as pharmaceutical excipients. In the tri-chamber architecture, the PVP crescent chamber serves as a fast-release carrier for DP, the hydrophobic round PLA core chamber acts as a sustained-release carrier for SH, and the drug-free round PLA sheath chamber provides spatial drug separation and structural support while regulating the release timing.

## 2. Materials and Methods

### 2.1. Materials

Polyvinylpyrrolidone (PVP K90, CAS: 9003-39-8, purchased from Sigma-Aldrich (Shanghai) Trading Co., Ltd., Shanghai, China); polylactic acid (PLA, CAS: 26100-51-6, particle size: 3 mm, Mw: 80,000); scopolamine hydrobromide (SH, CAS: 114-49-8, purchased from Hanzhong Hansuyuan Biotechnology Co., Ltd., Hanzhong, China); diphenhydramine (DP, CAS: 58-73-1, TCLD4744, 98.0% (GC&T), purchased from China National Pharmaceutical Group Chemical Reagent Co., Ltd., Shanghai, China); trifluoroethanol (TFEA); methylene blue; all chemical reagents were of analytical grade, and deionized water was used for all experimental water. MEM culture medium (10-010-CV, purchased from Shanghai Corning Management Co., Ltd., Shanghai, China); fetal bovine serum (164210-50, purchased from Wuhan Punosai Life Technology Co., Ltd., Wuhan, China); PBS (PB180327, purchased from Wuhan Punosai Life Technology Co., Ltd.); trypsin (BL512 A, purchased from Anhui Baisha Biotechnology Co., Ltd., Hefei, China); penicillin–streptomycin solution (100X, BL505 A, purchased from Anhui Baisha Biotechnology Co., Ltd.); and CCK8 (IV08-100, purchased from InCell Corporation, Irvine, CA, USA) were all provided by Scientific Compass (Shanghai, China).

### 2.2. Electrospun Preparation

The following working fluids were prepared for implementing the trifluid electrospinning processes to create the hierarchical structures.

(1) Crescent side solution: 12% (*w*/*v*) PVP K90, with the solvent composed of TFEA and deionized water at a volume ratio of 95:5. The specific preparation steps for the solution are to add 1.2 g of PVP K90 into a mixed solvent of 9.5 mL TFEA and 0.5 mL deionized water, making the total solvent volume 10 mL, and stir at 500 rpm at room temperature for 24 h.
(2) Circle sheath layer and core layer solution: 22% (*w*/*v*) PLA, with TFEA as the solvent. The specific preparation steps for the solution are to add 2.2 g of PLA into 10 mL of TFEA solvent, then heat it in a 55 °C oil bath at a stirring speed of 500 rpm for 24 h (heating promotes the dissolution of PLA while paying attention to preventing solvent evaporation caused by high temperature).
(3) Crescent side-drug-loaded solution: 12% (*w*/*v*) PVP K90 + 15% (*w*/*w*, DP/PVP) DP. After completely dissolving 12% (*w*/*v*) PVP K90, add 180 μL of oil liquid DP (having a density of 1.0 g/cm^3^) to the solution, and continue stirring for 6 h at room temperature in the dark.
(4) Circle core layer drug-loaded solution: 22% (*w*/*v*) PLA + 5% (*w*/*w*) SH. After the PLA is completely dissolved, add 0.11 g of SH to the solution and continue stirring for 6 h at room temperature in the dark.

The electrospinning device used in the experiment was self-assembled and mainly consisted of four parts: a high-voltage power supply, three syringe pumps, a homemade trifluid structural spinneret, and a piece of aluminum foil wrapped around a hardboard as the fiber collector. Through preliminary experiments, the trifluid electrospinning apparatus was used to prepare four types of nanofibers, namely samples S1, S2, S3, and S4. The components of working fluids and their spinning parameters are listed in Table 1. The environmental conditions were a temperature of 22 ± 3 °C and a relative humidity of 40 ± 5%.

### 2.3. Characterization of the Prepared Nanofibers

#### 2.3.1. Morphology

The morphology of electrospun nanofibers, including uniaxial nanofibers and core–shell nanofiber samples, was evaluated using a scanning electron microscope (SEM, Quanta FEG450, Hillsboro, OR, USA). A small piece of the fiber membrane was cut and fixed onto the sample stage with double-sided conductive adhesive; then, the sample was placed into the vacuum chamber. Prior to evaluation, the samples were gold-sputtered. Subsequently, the average diameter of the nanofibers at approximately 100 locations on the SEM images was estimated using ImageJ software.

#### 2.3.2. Internal Structure

The internal structures of uniaxial nanofibers and core–shell nanofibers were evaluated using a transmission electron microscope (TEM, JEM2200F, JEOL, Tokyo, Japan). During the electrospinning process, a 200-mesh copper grid was placed on the collector for less than 10 s to prepare the samples, and the collected nanofibers were placed in a vacuum chamber for examination.

#### 2.3.3. Physical State

Tests were conducted using an X-ray powder diffractometer (XRD, Bruker-AXS, Karlsruhe, Germany). All raw materials (PVP, PLA, DP, and SH) and the electrospun nanofiber membranes S1–S4 were measured over a 2θ angle range of 10–70°. The applied voltage and operating current were 40 kV and 30 mA, respectively.

#### 2.3.4. Compatibility

Fourier-transform infrared (FTIR) analysis was performed using a PerkinElmer FTIR spectrometer (Spectrum 100, Billerica, MA, USA) to study the compatibility between the polymer carrier and the active pharmaceutical ingredient. Samples were scanned in the range of 500–4000 cm^−1^, with a resolution of 2 cm^−1^. For powdered solid materials, 0.2 g of potassium bromide powder was taken during sample preparation, ground with approximately 10 mg of the sample, and the mixture was compressed into a solid tablet, which was then placed in the instrument for scanning. For liquid drug samples, a single drop of the liquid was applied to a blank potassium bromide tablet for measurement. For nanofiber membranes, a small piece of the film was cut and placed on the sample to perform the scan.

#### 2.3.5. Mechanical Testing

Using a standard dumbbell-shaped specimen mold, the nanofiber membranes S1–S4 were cut into test strips with a central width of 5 mm. Thickness measurements were taken at three points along the length of each strip using a spiral thickness gauge, and the average value was taken as the sample thickness. Tensile tests were conducted on the nanofiber membranes using a 2.5 kN universal testing machine, with a tensile rate set at 2.5 mm/min. Based on the experimental results, the tensile strength and elongation at break for each group of samples were calculated, and stress–strain curves were plotted. Three parallel specimens were tested in each experimental group, and the results were averaged.

#### 2.3.6. Wettability Testing

A surface tension meter was used to test the surface wetting properties of electrospun nanofiber membranes. First, the samples were cut into 50 × 10 mm^2^ rectangular specimens, mounted on microscope slides, and placed on the instrument’s testing platform. Deionized water was used as the test liquid, and contact angle measurements were performed using the static droplet method, with continuous image capture used to record the morphological changes of the droplet at different time points. Each set of samples was tested in triplicate, and the corresponding water contact angle values were calculated using ImageJ.

Meanwhile, a droplet of water was placed on the hierarchical fibers’ surface, which was collected on a glass slide for several minutes. An optical microscope was utilized to observe the dissolution behaviors of PVP sides. Pure PVP nanofibers were also prepared for comparison under the following experimental conditions: 12% (*w*/*v*) PVP K90 dissolved in a solvent mixture of TFEA/deionized water (*v*/*v*, 9.5:0.5), an applied voltage of 12 kV, a collection distance of 20 cm, and a stainless-steel tube with an inner/outer diameter of 0.51/0.82 mm (21G) as the spinneret.

#### 2.3.7. Quantitative Measurement, Drug Encapsulation Efficiency, and In Vitro Dissolution Tests

Establishment of the DP standard curve: 25 μL of oil liquid DP was accurately pipetted and dissolved in 250 mL of phosphate buffer solution (PBS, 0.01 M, pH 7.4 ± 0.2). The volumetric flask was protected from light. First, a DP stock solution with a concentration of 100 μg/mL was prepared, then it was progressively drawn out and diluted to obtain a series of low-concentration DP solutions (*C*, μg/mL). Using a UV–vis spectrophotometer, the absorbance (*A*) of each DP solution was measured at a maximum wavelength of 258 nm. The DP standard curve was obtained according to the Lambert–Beer law. The linear regression equation was *A* = 0.0019*C* + 0.0033 (R^2^ = 0.9986), within a linear range of 5 to 60 μg/mL.

Establishment of the SH standard curve: 25 mg of SH was accurately weighed and dissolved in 250 mL of PBS (0.01 M, pH 7.4 ± 0.2). First, an SH stock solution with a concentration of 100 μg/mL was prepared, and further dilutions were conducted to obtain SH solutions with various low concentrations (*C*, μg/mL). Their absorbances (*A*) were measured at 215 nm using a UV–visible spectrophotometer. The SH standard curve was obtained according to the Lambert–Beer law. The linear regression equation is *A* = 0.0123*C* + 0.001 (R^2^ = 0.9996), within a linear range of 2 to 22 μg/mL.

The drug encapsulation efficiency (EE%) was determined as follows. Accurately weighed amounts of the fibrous films S2 and S3 were dissolved in 10 mL of TEFA. Then, 1.0 mL of each solution was slowly dropped into 100 mL PBS under vigorous magnetic stirring. One milliliter of the resulting suspension was withdrawn, appropriately diluted with PBS, and filtered through a 0.22 µm MCE membrane (Navigator Lab Instrument Co., Ltd., Tianjin, China) before UV–Vis measurement. The encapsulation efficiency (EE%) was calculated using the following Equation (1):

(1) EE(%)=WmWt×100%

where W_m_ is the drug content determined in the fibrous films and W_t_ is the drug content added to the electrospinnable solutions.

In order to prevent mutual interference of DP and SH released from nanofibers S4, a combined strategy used in a previous study [9] was exploited, i.e., drug release of nanofibers S2 and S3 containing only DP and SH, respectively, were jointly analyzed for evaluating the asynchronous release profiles of DP and SH from nanofibers F4. To determine the drug release rates of core–sheath nanofibers, in vitro drug dissolution tests were conducted using the paddle-type method, as described in the 2015 edition of the Chinese Pharmacopoeia. An SHZ-86 water-bath constant-temperature shaker (Changzhou Jintan Shuibeike Pu Experimental Instrument Factory, Changzhou, China) was used to conduct the in vitro dissolution tests. Approximately 100 mg of the drug-loaded nanofiber membrane was immersed in 450 mL of PBS (0.01 M, pH 7.4 ± 0.2) at 37 °C and a rotation speed of 60 rpm. At predetermined time points, 4.0 mL of the supernatant was withdrawn, and 4.0 mL of fresh PBS was added. The absorbance was measured at λmax using a UV–vis spectrophotometer. The experiment was repeated six times. The cumulative percentage of drug release (*P*%) was calculated using Formula (2):

(2) $$P\%=\frac{c_{n}\times V_{o}+\sum_{i=1}^{n-1}c_{i}\times V}{Q_{o}}\times 100\%$$

Here, *V*_0_ is the volume of the elution medium (450 mL), *V* is the volume of the sample to be extracted (4 mL), *Q*_0_ is the theoretical amount of the drug in each sample (mg), C_n_ is the drug concentration measured in the nth aliquot (mg/L), and *C_i_* is the drug concentration measured in the i-th aliquot (mg/L).

#### 2.3.8. CCK-8 Assay for Cell Cytotoxicity Testing

The experiment consisted of three groups: control, S1, and S4. In the control group, 1 mL of complete culture medium was added to each well; in the S1 and S4 groups, circular sample slices with a diameter of 8 mm were placed in each well. Each group included three replicate wells. L929 cells in the logarithmic growth phase were selected, counted, and adjusted to a density of 5 × 10^4^ cells per well before being seeded into a 24-well plate. The cells were cultured overnight at 37 °C in a 5% CO_2_ incubator until they fully adhered to the well surfaces. After applying the treatments according to the predetermined groupings, the cells were incubated for 12 h and 48 h, respectively, under the same culture conditions. The original culture medium was aspirated, and each well was washed three times with PBS. One milliliter of culture medium containing 10% CCK-8 was added to each well, which was then incubated for 2 h in a 37 °C, 5% CO_2_ incubator. Finally, the absorbance at a wavelength of 450 nm was measured using a microplate reader.

(3) $$\mathrm{RCV}\%=\frac{A_{s}-A_{b}}{A_{c}-A_{b}}\times 100\%$$

In this experiment, *RCV*% represents the relative cell viability; *A*_s_ is the absorbance value for the experimental group; *A_b_* is the absorbance value for the blank group (containing only CCK-8 reagent and culture medium); and *A_c_* is the absorbance value for the control group (normally cultured L929 cells). All measurements were repeated three times.

#### 2.3.9. Cell Live/Dead Staining Experiment

This experiment consisted of three groups: control, S1, and S4. In the control group, 1 mL of complete medium was added to each dish; in the S1 and S4 groups, a 10 mm-diameter sample disc was added to each dish; L929 cells in the logarithmic growth phase were selected, counted, and adjusted to a concentration of 1.5 × 10^5^ cells per dish before being seeded into confocal culture dishes. The dishes were incubated overnight in a constant-temperature incubator at 37 °C and 5% CO_2_ until the cells adhered to the surface. After treatment according to the group assignments, incubation was continued for 12 h and 48 h. Staining solutions were prepared fresh for each use: Reagent A (Calcein-AM) and Reagent B (PI) were each diluted 10-fold with dye diluent (Solution C), then mixed thoroughly with PBS. First, the cells were washed once with PBS to remove excess serum. Then, 1 mL of staining solution was added to each dish, and the dishes were incubated at room temperature in the dark for 15 min. After staining was terminated by washing three times with PBS, the results were observed under a microscope, and images of the fields of view at 100× and 200× magnification were captured.

### 2.4. Statistical Analysis

All experiments were performed with at least three independent repetitions, and the experimental data are expressed as mean ± standard deviation. Statistical significance was assessed using one-way analysis of variance (ANOVA) with Tukey’s test for multiple comparisons (GraphPad Prism software version 6.0). *p* < 0.01 was considered to indicate a statistically significant difference between groups.

## 3. Results and Discussion

### 3.1. The Trifluid Electrospinning

**Figure 1:**
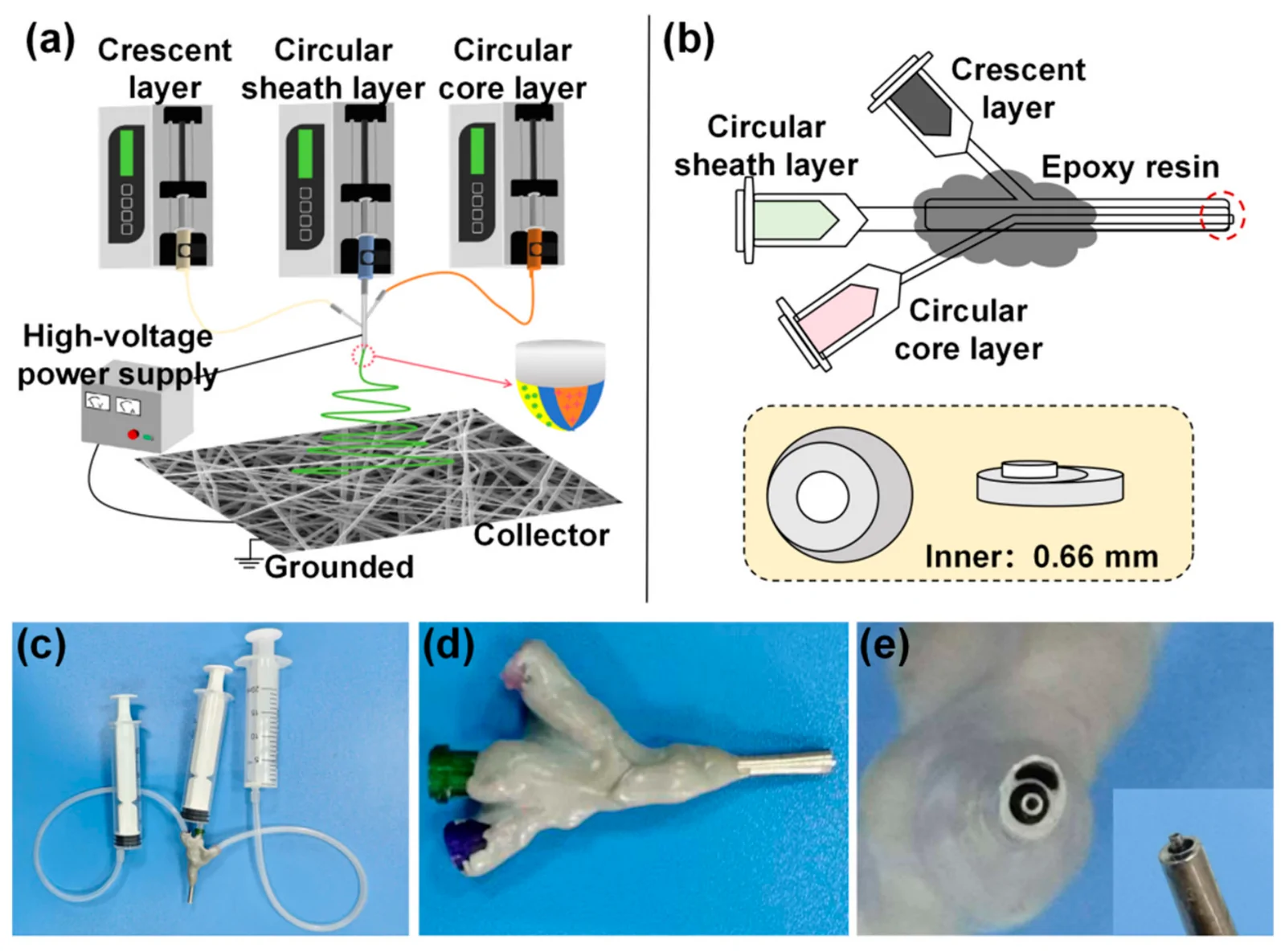
Trifluid electrospinning technique: (**a**) a schematic diagram of the spinning process; (**b**) internal structure of the spinneret; (**c**) connection between the spinning head and the three syringes; (**d**) photograph of the spinning head; (**e**) view of the spinning head nozzle; the bottom-right inset shows a side view of the nozzle.

In this study, a custom-made structural spinneret containing three channels and a corresponding electrospinning apparatus were prepared for implementing the trifluid electrospinning processes for creating the tri-chamber hierarchical nanostructures. The main difference between this setup and a traditional popular coaxial electrospinning setup lies in the spinning head, which is also the key factor in forming multi-chamber nanofibers. The design and preparation details of the structural spinneret are shown in Figure 1. Figure 1a shows a simplified schematic diagram of the trifluid electrospinning apparatus and working process. Figure 1b shows the internal structure of the spinning head. The two needles on the circular side share a common shaft; as seen from the side view, the needle for the core layer protrudes slightly, by about 0.6 mm, which effectively guides the inner core fluid and prevents its potential diffusion. The crescent-shaped side and the sheath chamber of the round side form an inscribed relationship. All the metal capillaries combine at one end to form the structural nozzle of the spinneret, first side-by-side and second with one side, showing a core–sheath hierarchic configuration. Each of their other ends is connected with a plastic slot for facile connection of a syringe or silicon tube, as indicated in Figure 1c. The crescent-shaped side and the core layer of the circular side of the spinning head are connected via a silicone tube and a syringe, while the sheath channel of the circular side is directly connected to the syringe. The connections of plastic with metal capillaries are primarily secured using epoxy resin. For detailed images of the actual spinneret, refer to Figure 1d,e. The whole organization of the spinneret is compact, with a slight weight of about 8.7 g (Figure 1d). The complex macro hierarchical structures are clear in Figure 1e; the bottom-right inset shows a side view of the nozzle.

**Figure 2:**
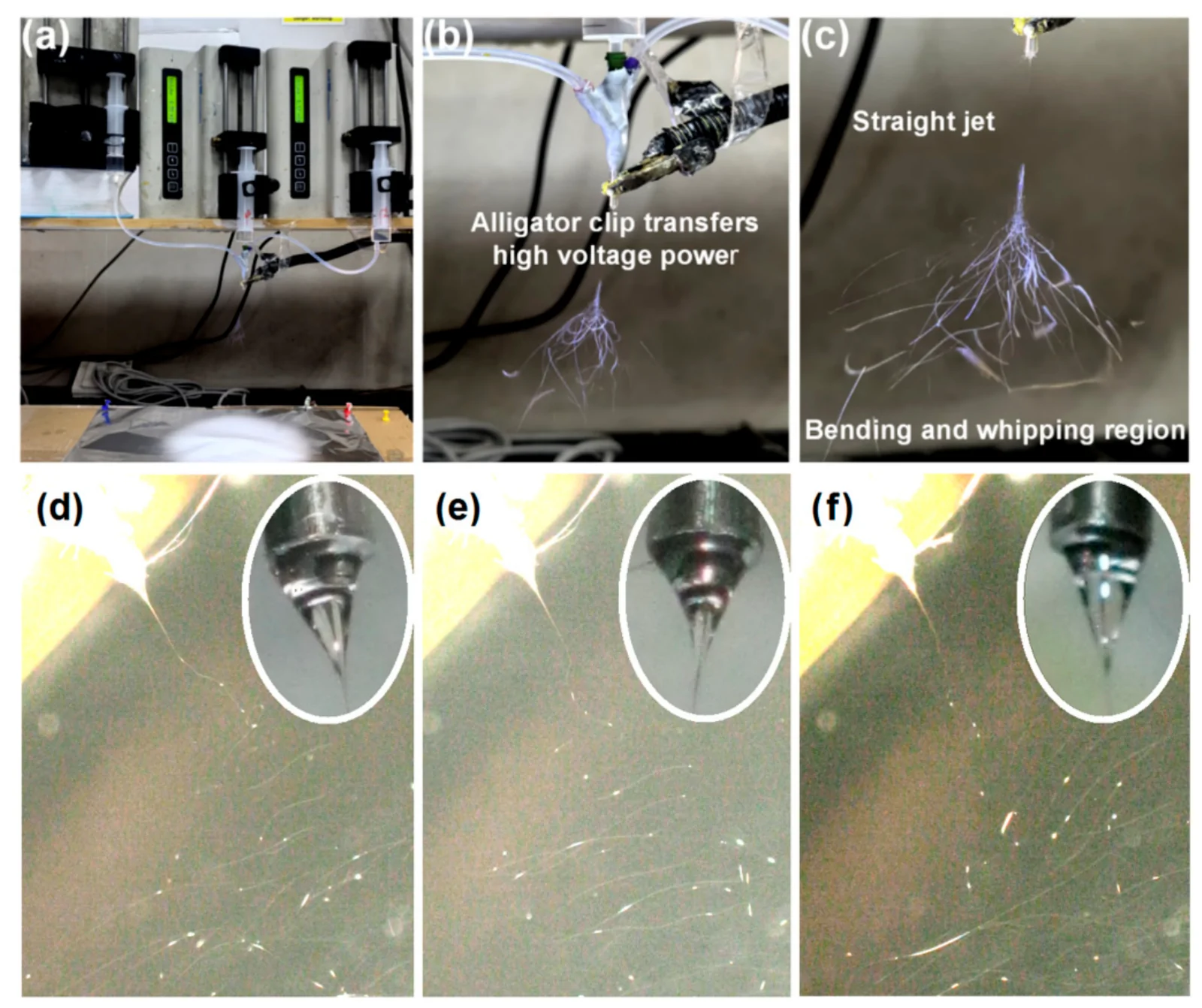
Diagrams of the experimental process for the three-fluid parallel coaxial composite electrospinning technique: (**a**) schematic diagram of the spinning apparatus; (**b**) high-voltage power supply connection; (**c**) image of the solution stretching and solidification process captured with a cell phone; (**d**–**f**) the real bending and whipping processes in the unstable region taken from various angles; the upper-right insets are the corresponding compound Taylor cones.

The self-assembled electrospinning setup is shown in Figure 2a. The three syringe pumps primarily used drive the solutions for the crescent channel, the circular core channel, and the sheath channel, respectively. The positive terminal of the high-voltage power supply is connected to the needle and its negative terminal is connected to the collector, which is securely grounded. The positive terminal is primarily attached to the spinning head via an alligator clip, utilizing the conductive properties of metal to apply high voltage to the solution (Figure 2b). Grounding the negative terminal serves to ensure the safety and stability of the equipment, protect personnel, and minimize interference from high-voltage frequencies during the experiment. Under the influence of the high-voltage electric field during electrospinning, the solution travels from the tip of the Taylor cone to its final deposition on the collector, passing through four main regions: the Taylor cone, the straight jet, the curved whipping region, and the solidified fiber. Figure 2c clearly shows the straight jet and the curved whipping process, and contains visual illusions of continuous divisions. This is due to the poor capture capability of the Huawei Smart phone. A Canon camera (GX7, Canon, Tokyo, Japan) was exploited to take photos of working processes; these images are shown in Figure 2d–f, and were taken from different angles. The typical three successive steps of traditional blending electrospinning are obvious, i.e., Taylor cone, straight fluid jet, and the bending and whipping regions [42,43,44,45]. The compound Taylor cones formed by the three working fluids under the applied high voltage are shown in the upper-right insets of Figure 2d–f.

### 3.2. Morphology and Inner Structures

**Figure 3:**
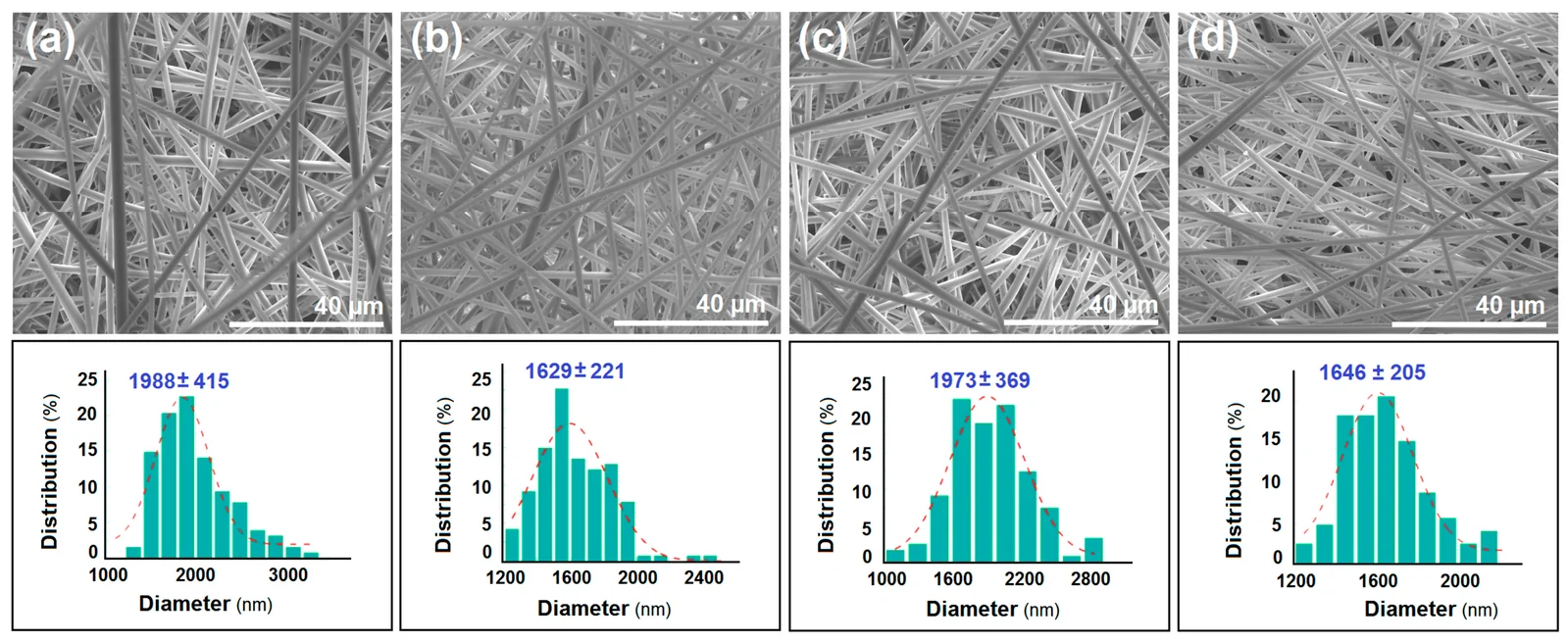
SEM images and diameter distribution histograms of electrospun nanofibers: (**a**) S1; (**b**) S2; (**c**) S3; (**d**) S4.

Figure 3 shows the SEM morphologies and statistical results of the diameter distributions for four sets of nanofibers with a “PVP crescent chamber–PLA sheath chamber–PLA core chamber” segmented structure, prepared using a trifluid guiding structural spinneret. SEM images reveal that all the four sets of samples exhibit a continuous, bead-free, smooth-surfaced, three-dimensional interlaced porous network structure with no visible agglomeration. There were no interface fusion, structural collapse, or morphological defects resulting from multifluid co-spinning. Sample S1 had an average diameter of 1988 ± 415 nm, representing fibers produced without drug interference. The average diameter of S2 was 1629 ± 221 nm, a significant reduction compared to S1. This was primarily due to DP, a small-molecule drug; its introduction into the PVP crescent layer reduced the solution’s surface tension and increased its electrical conductivity. At the same time, the optimization of physicochemical properties significantly improved the stability of the spinning process, suppressed unstable fluctuations in the jet, and resulted in a more concentrated fiber diameter distribution with markedly enhanced uniformity. The average diameter of S3 was 1973 ± 369 nm, which was highly consistent with that of S1, showing no obvious difference. This is because SH was loaded only in the innermost PLA core layer and did not alter the rheological or conductive properties of the outer two-phase layer; therefore, it had no significant effect on the jet’s whiplash stretching behavior. The average diameter of S4 was 1646 ± 205 nm, showing no significant difference from S2. These results indicate that the introduction of SH into the core layer did not interfere with the regulatory effect of DP in the crescent layer on the spinning process. This indicates that the three-fluid organizations have the ability to load the two drugs independently without mutual interference, thereby completely avoiding the deterioration of spinning solution properties and structural defects that might result from blending the two drugs. At the same time, the co-loading of the two drugs not only did not disrupt the fiber structure but, through the regulatory effect of DP, actually optimized the microstructure of the fibers; finer and more uniform nanofibers provide a larger specific surface area and a denser porous network. Additionally, although the nanofibers have a diameter larger than 1 micron, their three chambers are still smaller than 1 micron; based on this fact and the conventional parlance, we still refer to these fibers as nanofibers.

**Figure 4:**
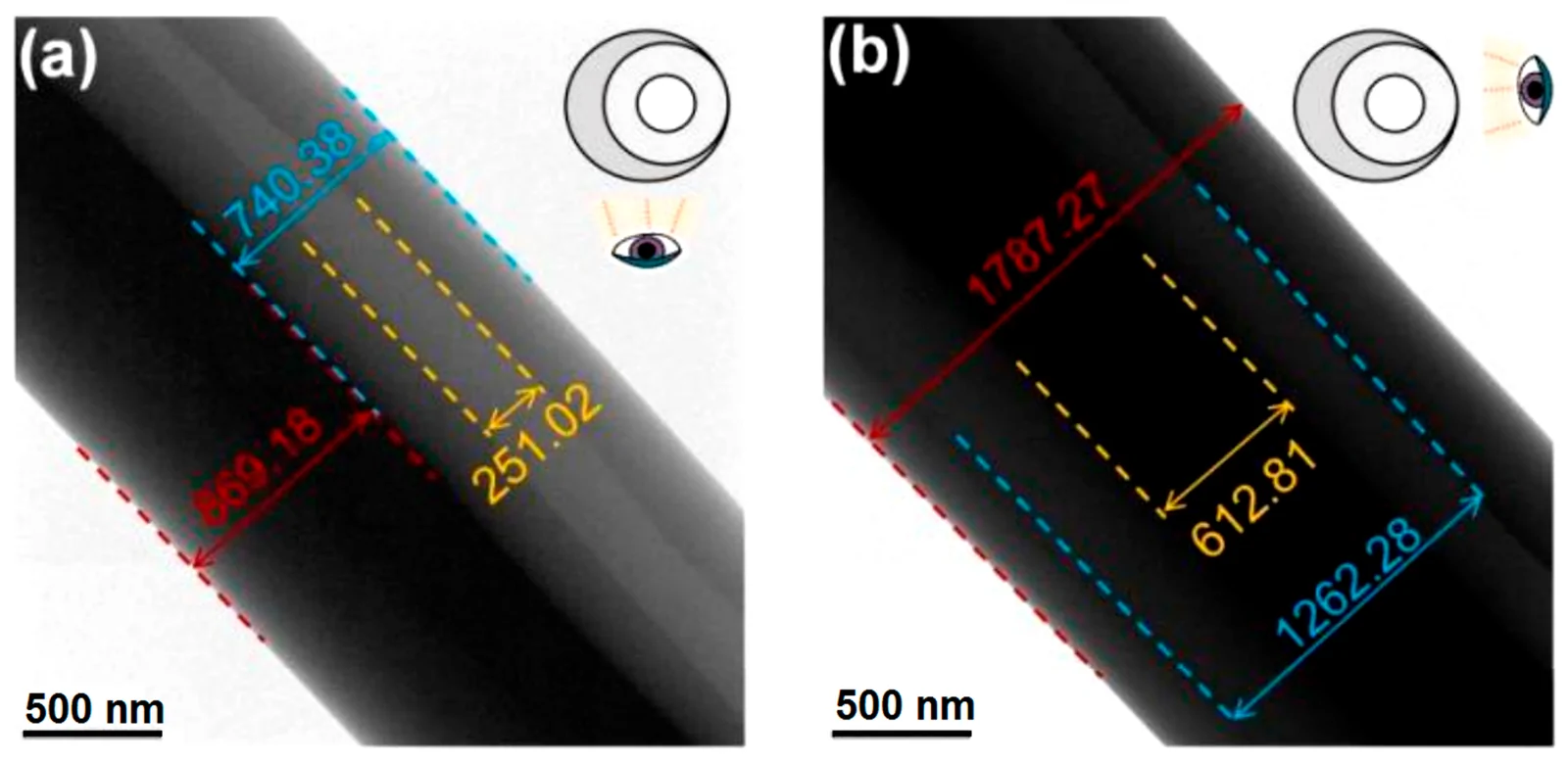
TEM images of the S4 nanofiber membrane taken from different angles: (**a**) front view; (**b**) side view.

TEM characterization results (Figure 4) clearly confirm that the trifluid electrospinning technique can successfully prepare dual-drug-loaded nanofiber membranes with a core–sheath–crescent hierarchical configuration. In the head-on view (Figure 4a), individual fibers exhibit a distinct three-layer hierarchical structure: the circular lateral core layer consists of a PLA matrix loaded with SH, with a thickness of 251.02 nm; the intermediate sheath layer consists of a pure PLA support layer, with a 740.38 nm diameter; and the outer crescent-shaped layer consists of a PVP matrix loaded with DP, with an 869.18 nm thickness. The interfaces between the layers are clear and sharp, with no obvious intermixing of components or structural defects, indicating that the fluids achieved stable layered solidification during the electrospinning process. A side view (Figure 4b) further verified the integrity of the fiber’s three-dimensional structure. The total diameter of the nanofiber was measured to be 1787.27 nm, with the transverse dimensions of the sheath and core layers being 1262.28 nm and 612.81 nm, respectively. These results demonstrate that the electrospinning strategy proposed in this study—which involves the electrospinning of nanofibers with a three-fluid parallel-encapsulated complex structure—can precisely control the spatial distribution of multicomponent materials, providing a solid structural foundation for the differential loading and controlled release of two drugs.

The microformation mechanism of the tri-chamber structures from trifluid electrospinning is important for robust and continuous production. The successful creation of these structures relies on two key factors. One is the well-known capability of electrospinning to readily replicate complex nanostructures using the spinneret nozzle as a template [46,47,48]. This is also why the spinneret is regarded as the most important part of an electrospinning system and has been a focus of continuous innovation [49,50]. The other is that mutual diffusion between the two contacting working fluids is possible; however, it can be neglected as long as the two fluids remain parallel to each other during the fast drying process [51].

### 3.3. Physical Properties and Compatibility of Components Within the Nanofibers

**Figure 5:**
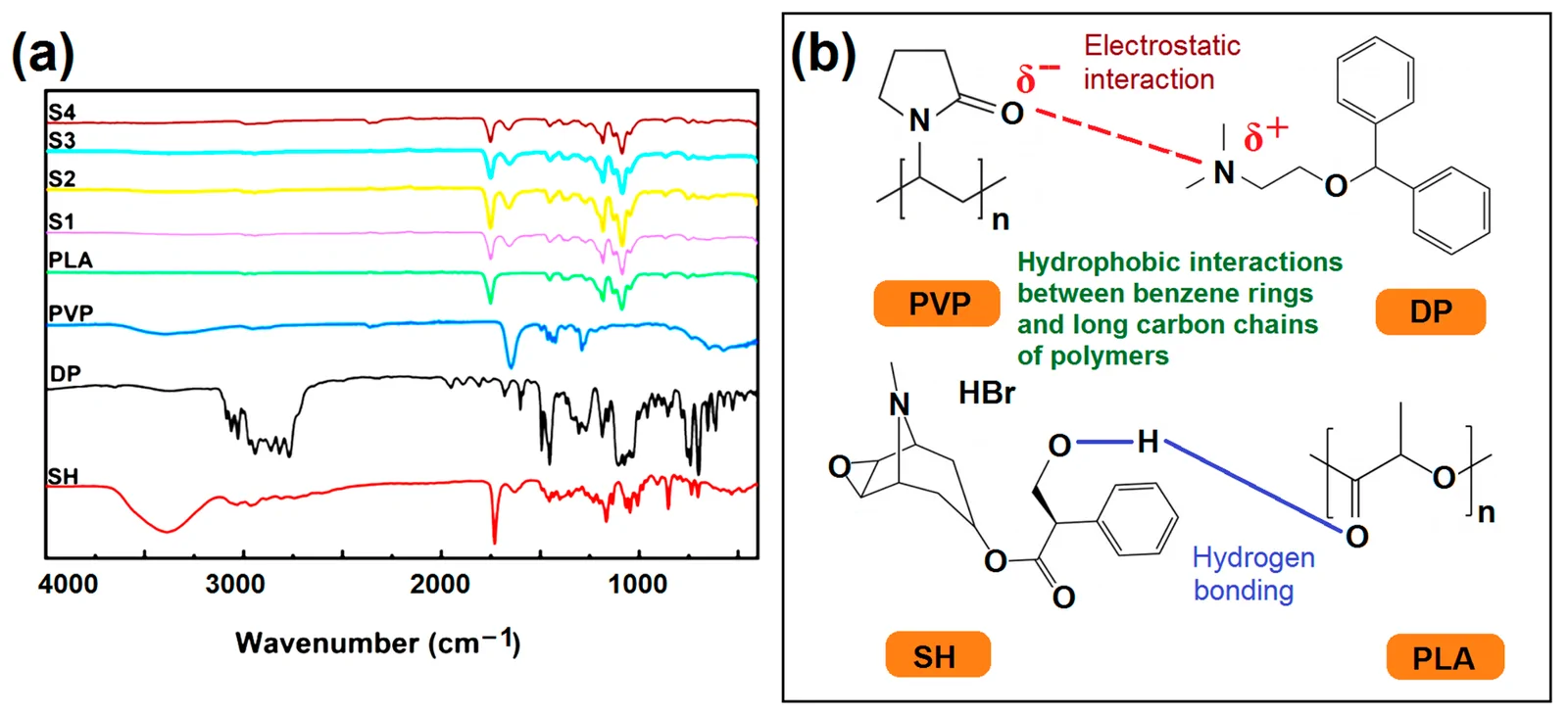
ATR-FTIR spectra of raw materials of PVP, PLA, SH, and DP, as well as their nanofibers S1–S4 (**a**) and the chemical structures of the raw components (**b**).

Figure 5 shows the ATR-FTIR spectra of PVP, PLA, the pure drug DP, SH, and the four sets of nanofibrous membranes (S1–S4). The spectral characteristics show that all four types of nanofibrous membranes fully retained the core characteristic peaks of PVP and PLA, and the introduction of the drug did not induce new absorption peaks from off-target groups, directly confirming the chemical compatibility. The characteristic PVP peaks at 1660 cm^−1^ and 1290 cm^−1^ were clearly present in nanofibers S1–S4, with no significant distortion in peak shape, indicating that the PVP side maintained its molecular structural integrity during the spinning process. The characteristic PLA absorption peaks at 1748 cm^−1^ and 1180 cm^−1^ were strong in all fiber membranes, proving the co-presence of this hydrophobic polymeric matrix with PVP in all the nanofibers. Comparing the spectra of the pure drug with those of the fiber membranes, the characteristic absorption peaks of DP around 3000 cm^−1^ and 600 cm^−1^, and also those in the fingerprint region, were significantly reduced or even completely disappeared in the spectra of S2 and S4, but no new interference peaks appeared. These phenomena should be attributed to the small contents of DP in the nanofibers and also the favorable secondary interactions between DP and PVP molecules (such as the hydrophobic interactions and the electrostatic interactions, Figure 5b) in the electrospun fibrous nanocomposites. Similarly, the characteristic SH peaks at 3370 cm^−1^ and 1727 cm^−1^, and those fingerprint region’s peaks, have very small or even no absorbances in the spectra of nanofibers S3 and S4, and new interference peaks also did not appear. It is the favorable secondary interactions (such as hydrogen bindings and hydrophobic interactions, Figure 5b) between SH and PLA molecules that result in the reduction in and even disappearance of SH’s peak absorbance from the spectra of nanofibers S3 and S4. Those secondary interactions are favorable for the good compatibility between the drug and its polymeric carrier molecules, which are in turn favorable for the physical stability of the electrospun fibrous nanocomposites.

**Figure 6:**
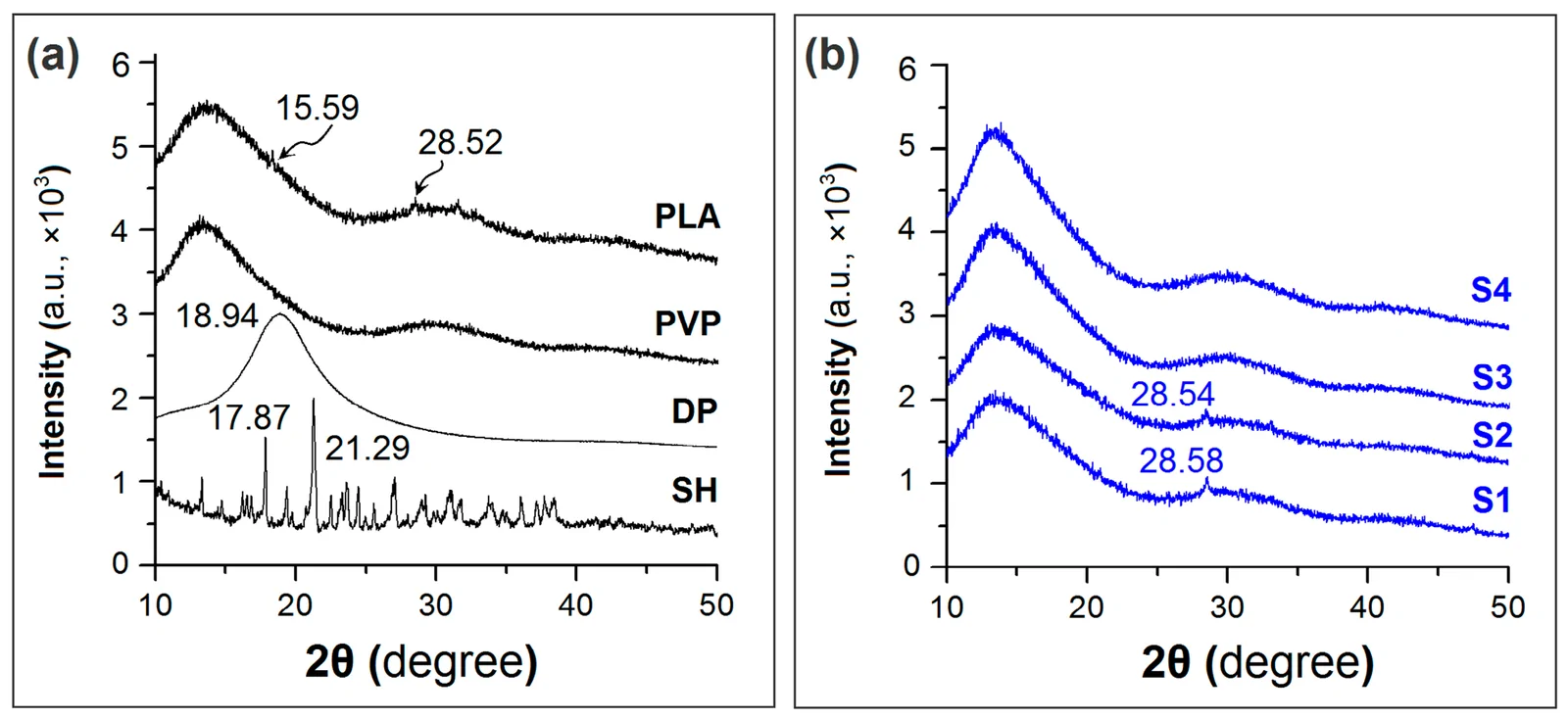
XRD patterns of (**a**) raw materials of PVP, PLA, SH, and DP, and (**b**) their hierarchical nanofibers S1–S4.

Figure 6 shows the XRD patterns of PVP, PLA, pure DP, SH, and four kinds of tri-chamber nanofibrous membranes (S1–S4), which were used to systematically analyze the crystalline structure and drug loading status of the materials. As shown in the patterns of the raw materials in Figure 6a, pure SH exhibits a series of sharp (such as 2θ = 17.87° and 21.29°) high-intensity characteristic diffraction peaks, indicating that it is a typical crystalline drug. The pure DP shows only a broad, diffuse “doughnut-shaped peak” at 2θ = 18.94°, with no sharp crystalline phase peaks, suggesting that DP has an amorphous raw state. PVP exhibits an amorphous state. It displays two typical broad halos. PLA exhibits a typical semi-crystalline state, with a characteristic broad halo and two small sharp peaks at 2θ = 15.59° and 2θ = 28.52°due to the α-phase.

A comparison of the XRD patterns of the four types of nanofibrous membranes shows that the diffraction characteristics of S1 are consistent with those of pure PVP and PLA, retaining the broad, diffuse peaks of the polymers and the small sharp peak at 2θ = 28.58° owing to the α-phase, without the appearances of any new crystalline phases (Figure 6b). This confirms that the three-fluid spinning process did not alter the crystalline structure of the matrix polymers. The XRD pattern of S2 almost completely overlaps with that of S1 with the small sharp peak at 2θ = 28.54°, with no additional characteristic peaks appearing. Due to the conversion processes of similar components from similar materials, these amorphous polymer-based nanocomposites often have high similarity in their XRD patters [52,53,54,55]. The pattern of S3 also exhibits only broad, dispersed peaks of PVP and PLA, with the sharp crystalline peaks characteristic of pure SH completely absent. This result suggests that, during the electrospinning process, the SH loaded onto the PLA core layer was transformed from a crystalline state to an amorphous state. Meanwhile, the small peaks around 2θ = 28.52° to 28.58° appearing in the patterns of PLA, S1 and S2 also completely disappeared. These phenomena should be attributed to the secondary interactions between SH and PLA molecules, as mentioned above. Meanwhile, it should also be noted that the very small contents of crystal materials may not be detected. XRD has a poor detection accuracy when the contents are smaller than 5% [56,57]. As anticipated, the XRD pattern of S4 showed no sharp characteristic peaks of SH or the small sharp peak of PLA, retaining only the broad, diffuse peaks of the polymers, indicating the amorphous state of drugs within their nanocomposites.

The crystalline state is a low-energy physical state of matter. In particular, the particle sizes of drug crystals vary considerably, and the lattice energy must be overcome during dissolution. These factors make the dissolution and release of drugs from conventional formulations—such as tablets made by powder compression—difficult to predict and precisely control. In contrast, when drug molecules are distributed in an amorphous/molecular state within a polymer matrix, their release can be governed by the swelling, dissolution, or degradation behavior of the polymer. This therefore facilitates accurate prediction and precise controlled release of the drug, enabling safe and effective drug delivery. Electrospinning, whether single-fluid or multi-fluid, can instantaneously dry the spinning solution, thereby preserving the highly uniform dispersion state of the drug in the spinning solution and transferring it into the nanofibers. This yields amorphous composite nanofibers, and consequently, electrospinning has been widely applied to the dissolution enhancement and controlled release of poorly water-soluble drugs [52].

### 3.4. Mechanical Testing

**Figure 7:**
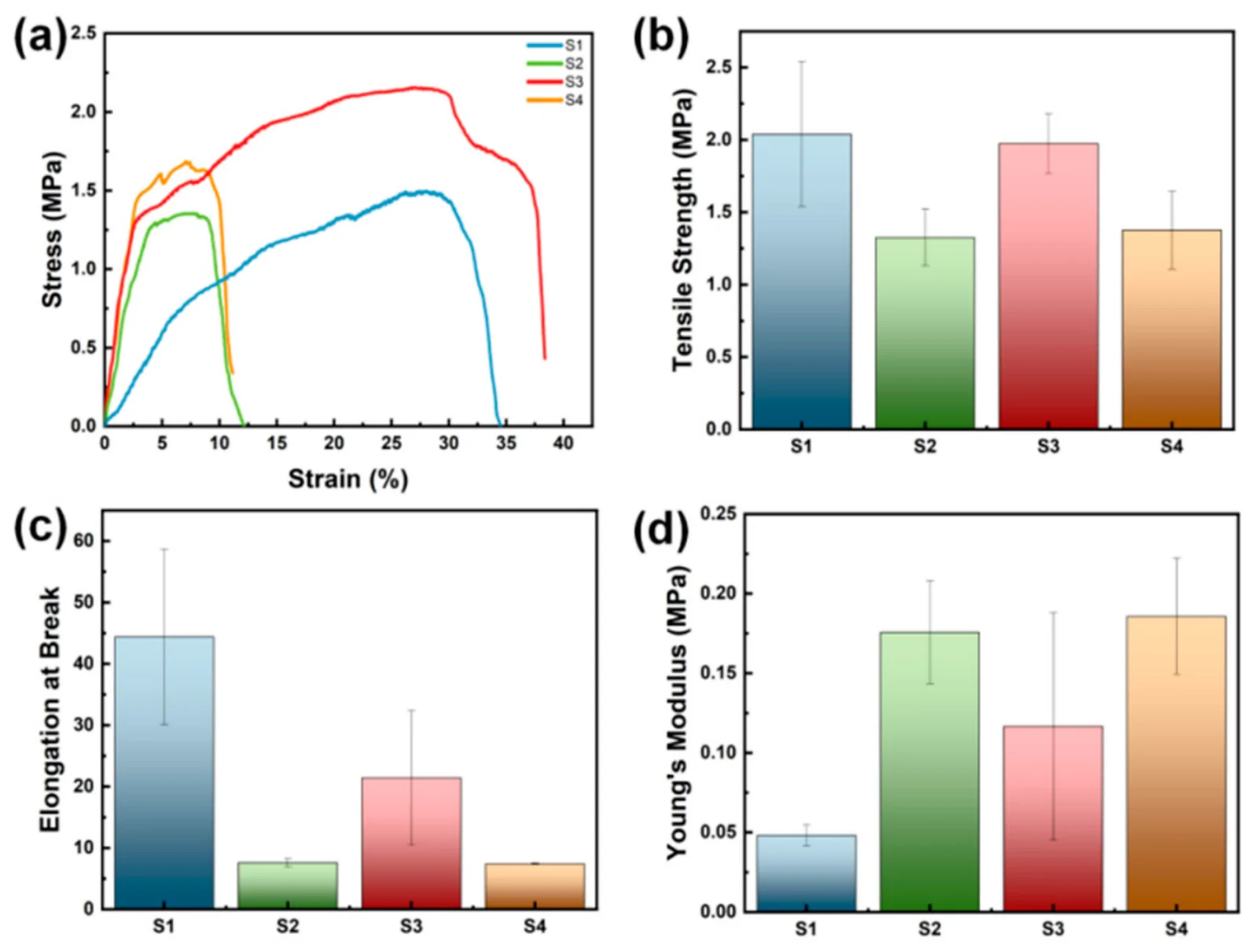
Mechanical properties of nanofibers S1–S4: (**a**) stress–strain curves; (**b**) tensile strength; (**c**) elongation at break; (**d**) Young’s modulus.

Figure 7 systematically characterizes the mechanical properties of four kinds of nanofibrous membranes (S1–S4), including stress–strain curves (Figure 7a), tensile strength (Figure 7b), elongation at break (Figure 7c), and Young’s modulus (Figure 7d). It comprehensively evaluates the mechanisms by which drug loading modulates the mechanical behavior of the fiber membranes, as well as the mechanical suitability of the material for use as an oral mucosal patch. As shown in the stress–strain curves, all four groups of samples exhibited typical tensile behavior of flexible polymers, undergoing an initial elastic stage followed by a plastic yield stage before fracture, with no signs of brittle fracture. This aligns with the flexible substrate characteristics required for oral films. Quantitative statistical results show that the mechanical properties of each group exhibit significant drug-load dependence: S3 had the highest tensile strength (1.98 MPa), with no significant difference from S1 (2.05 MPa), indicating that the introduction of the SH core layer did not disrupt the continuous phase structure of the PLA matrix and that intermolecular forces enhanced fiber entanglement, thereby maintaining excellent structural strength. The tensile strengths of S2 and S4 were 1.35 MPa and 1.42 MPa, respectively; although slightly lower than that of S1, they still met the clinical requirements for oral films [58,59]. This reduction in strength was attributed to the weakening effect of DP on the hydrogen bonds between PVP chains. In terms of elongation at break, S1 exhibited the best toughness, with an elongation at break of 45.2%, while the drug-loaded groups S2, S3, and S4 showed elongation at break values that decreased sequentially to 7.8%, 21.8%, and 5%, This phenomenon is attributed to the restrictive effect of small-molecule drugs on polymer chain motion: DP loaded into the PVP crescent layer and SH loaded into the PLA core layer each inhibit the slip ability of the molecular chains in their respective phases. When both drugs are co-loaded, this effect is cumulative, leading to reduced film extensibility and increased rigidity. The Young’s modulus results further corroborate this pattern: S4 exhibited the highest Young’s modulus (0.19 MPa), followed by S2 (0.18 MPa) and S3 (0.12 MPa), with S1 having the lowest value (0.05 MPa), indicating that drug loading can significantly increase the film’s stiffness and enhance structural stability. Overall, the mechanical properties of all four sample groups meet the application requirements for oral mucosal patches. Although the dual-drug-loaded group S4 exhibits slightly reduced extensibility, it still meets clinical requirements and possesses the highest structural stability.

### 3.5. Wettability Testing

**Figure 8:**
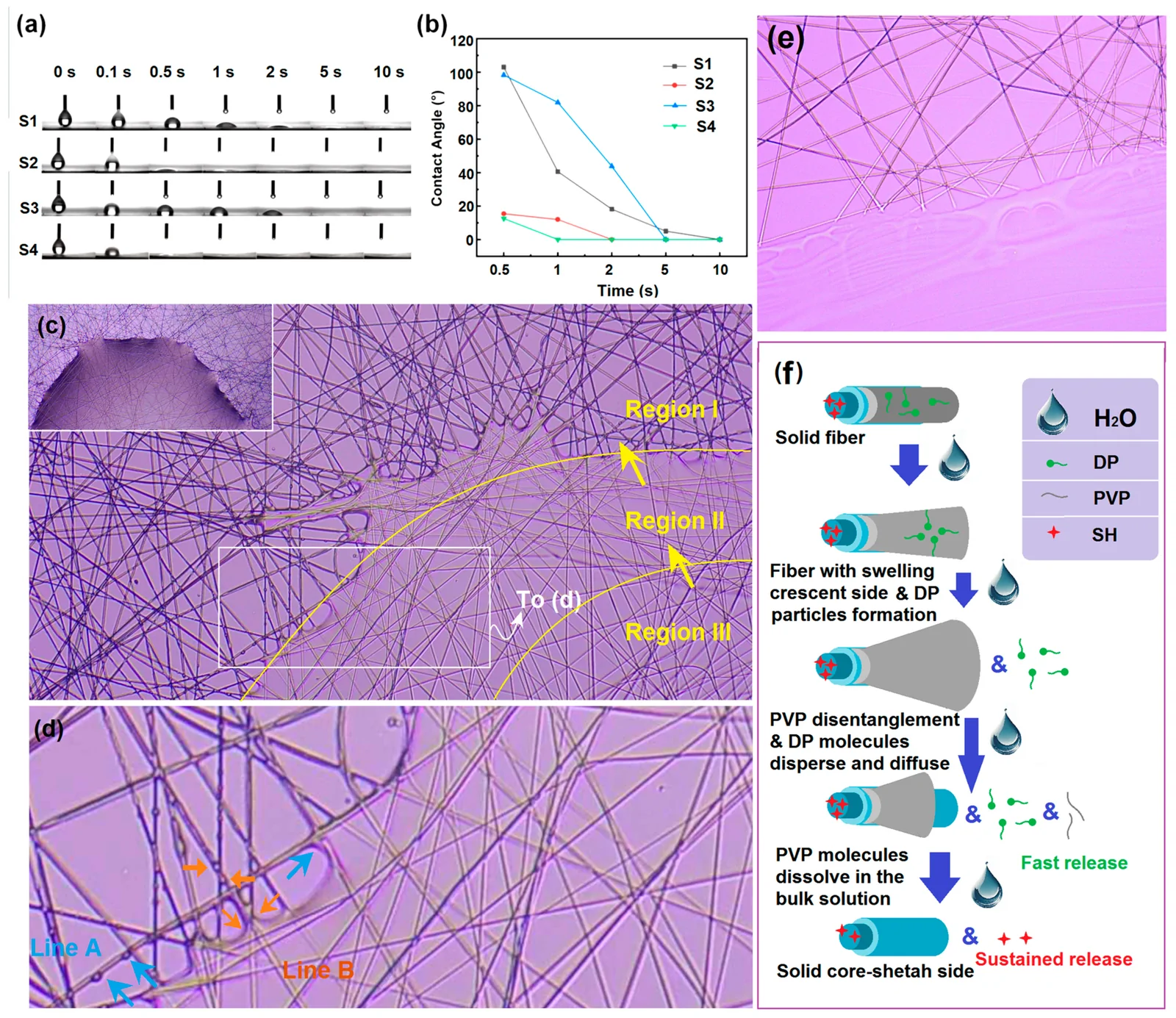
(**a**) Observation of the dynamic changes in a drop of water placed on the surface of the S1–S4 nanofiber membranes over a 10 s period; (**b**) curve showing the trend in WCA angle changes; (**c**) a section view when a droplet of water was dripped on the nanofibrous films collected on a glass slide (with a magnification of 100×); the upper-left inset shows a half image of the water droplet (with a magnification of 40×); (**d**) an enlarged image of the boundaries of the water droplet crawling along the hierarchical nanofibers (with a magnification of 400×); (**e**) an optical image of the boundaries of the water droplet crawling along the pure PVP nanofibers (with a magnification of 400×); (**f**) molecular mechanisms of the fast release of DP and sustained release of SH from the tri-chamber hierarchical structures.

For oral mucosal patches, excellent initial hydrophilicity and rapid wetting properties can significantly enhance the immediate adhesion between the membrane and the oral mucosa, accelerate drug dissolution and trans-mucosal absorption, and simultaneously improve patient comfort. In this study, dynamic water contact angle (WCA) testing was used to systematically evaluate the surface wettability and wetting kinetics of three-fluid electrospun nanofiber membranes under core–sheath structures and in drug-loaded states. Figure 8a shows the spreading and penetration process of a water droplet on the membrane surface over 10 s, while Figure 8b presents the trend curve of WCA over time, which was used to evaluate the material’s hydrophilic and hydrophobic properties and wetting behavior. As observed from the dynamic spreading process and the WCA trend curve, the wetting behavior of the four sample groups exhibited significant component dependence: S1 had an initial WCA of approximately 104°, exhibiting typical hydrophobicity; subsequently, the WCA decreased rapidly over time, dropping to about 5° at 5 s and achieving complete wetting (WCA of approximately 0°) at 10 s. This indicates that although the S1 membrane was initially hydrophobic, the hydrophilic components of the PVP crescent layer drove rapid water molecule penetration, resulting in rapid wetting. S2 had an initial WCA of approximately 16°, demonstrating excellent initial hydrophilicity; the WCA dropped to 0° within 2 s, making it the fastest-wetting group among all samples. This phenomenon stems from the introduction of DP, which further enhanced the hydrophilicity of the PVP crescent layer. Combined with the SEM results discussed earlier, DP caused the fibers to become finer and increased their specific surface area, providing more abundant penetration pathways for water molecules and accelerating the wetting process; S3 had an initial WCA of approximately 98°, similar to the initial hydrophobicity of S1. Its WCA decline rate was significantly slower than that of S1; it remained at approximately 45° at 5 s and was fully wetted by 10 s. This indicates that the introduction of SH restricted the penetration of water molecules into the core layer, thereby delaying the wetting process; S4 had an initial WCA of approximately 13°, which was highly consistent with the initial hydrophilicity of S2. The WCA dropped to 0° within 1 s, exhibiting ultra-fast wetting characteristics comparable to those of S2. This demonstrates that, when both drugs are co-loaded, the hydrophilic regulatory effect of DP in the crescent layer dominates, completely offsetting the delaying effect of SH in the core layer, while retaining the structural advantages of the dual-drug compartmentalized loading.

PVP, as a hydrophilic polymer and one of the most common pharmaceutical excipients for enhancing the dissolution of hundreds of poorly water-soluble drugs [60,61,62], is distributed in the crescent-shaped outer layer. This is the key factor determining the membrane’s initial hydrophilicity and wetting rate. PLA, as a hydrophobic polymer, forms the sheath and core layers, providing structural support and a sustained-release barrier for the membrane. DP, loaded into the PVP crescent layer, significantly enhances the outer layer’s hydrophilicity while refining the fiber structure and substantially accelerating wetting. SH is loaded only into the PLA core layer without altering the outer layer’s composition, and therefore has no significant effect on the initial WCA, merely delaying water molecule penetration through the barrier effect of the core layer. These WCA results fully validate the structural design advantages of three-fluid spinning. The ultra-rapid wetting characteristics of S2 and S4 perfectly align with the clinical need for “rapid onset of action” in the treatment of motion sickness, while the initial hydrophobicity of S1 and S3 delays drug release, making them suitable for long-acting prophylactic applications.

When an optical microscope was exploited to observe the dripping of a water droplet with a volume of about 3.0 µL on the collected nanofibrous membrane S4, a series of interesting phenomena were observed (Figure 8c,d). Firstly, the water scattered very rapidly, and the density of nanofibers determined the speed: the larger the density, the faster the creeping of the water droplet’s boundary. The upper-left inset of Figure 8c shows a half image of the water droplet (with a magnification of 40×). Further enlarging one section, shows three regions. The diameters of nanofibers have a size order of Region I > Region III > Region II. The former case is due to the swelling of nanofibers. The second case is because of the refraction effect of light under a water droplet. The smallest of the Region II fibers were obvious because they have only one core–sheath PLA side and thin water cover, resulting in less light refraction. Further enlarging the white rectangular section, and with nanofibers Line A and Line B as the typical samples, the dissolution of PVP-DP sides can be disclosed in detail.

Pure PVP K90 nanofibers were prepared and collected on a glass slide. A drop of water was dropped onto the nanofibers, and the resulting image is shown in Figure 8e. It is obvious that the pure PVP nanofibers dissolved very rapidly, with the swollen “enlarged” ends located close to the droplet region but without any separated particles. In the hierarchically structured nanofibers S4, the PLA-SH core–sheath sides did not dissolve in water. In contrast, the PVP K90 matrix of the PVP-DP sides dissolved rapidly upon contact with water, owing to its high hygroscopicity and water solubility. The loaded DP molecules, as a lipid oil substance, were released from the crescent PVP-DP nanocomposites when the composite sides swelled, leaving sufficient space for the movement of the DP molecules that had previously been homogeneously distributed and sealed within the PVP K90 matrix. These DP molecules would further aggregate into tiny particles owing to the hydrophobic attraction between DP molecules and the repulsion from the surrounding hydrophilic water molecules within the swollen PVP crescent (Figure 8d). As the PVP molecules in the further-swollen crescent sides dissolved into the bulk aqueous solution, the aggregated tiny DP droplets would gradually dissolve into the dissolution medium, since ample aqueous solution was provided, as indicated by the proposed mechanism in Figure 8f. This should be also be the reason why the tiny particles only transiently existed in those nanofibers in Region I and were close to Region II. There were no particles in Region III or the solid hierarchical nanofibers.

As indicated by the blue arrows, the two tiny particles and the swollen region are toward the same direction, suggesting the PVP-DP sides of the hierarchical nanofibers Line A. In contrast, the hierarchical nanofibers Line B should have the PVP-DP side upward, as indicated by the two orange arrows in the symmetrical swollen regions, and the two particles also vertically assembled on the residue PLA sides. These protocols provide a useful tool for further investigating some special molecular self-assembly based on hydrophilic–hydrophobic interactions, and also for optimizing the preparation parameters of structural spinnerets, such as the inner/outer capillaries’ size ratio, the projection of the inner capillary over the outer capillary, and the textures (metal or plastic) on the formation ratios of core–sheath nanostructures, which will never be 100%.

### 3.6. Analysis of In Vitro Drug Release

The DP EE% in nanofibers S2 and the SH EE% in nanofibers S3 were 97.87 ± 1.35% and 99.24 ± 0.62%, respectively. Electrospinning is an extremely fast physical drying process, during which not only are almost all the drugs encapsulated within the solid nanofibers with very limited loss [31], but the homogeneous state of drug and polymer in solution is also propagated into the fibers, forming uniform medicated nanocomposites. Furthermore, the blank PLA sheath layer was favorable for the complete encapsulation of SH within the core matrix, by preventing the possible diffusion and escape of SH molecules into the environment.

**Figure 9:**
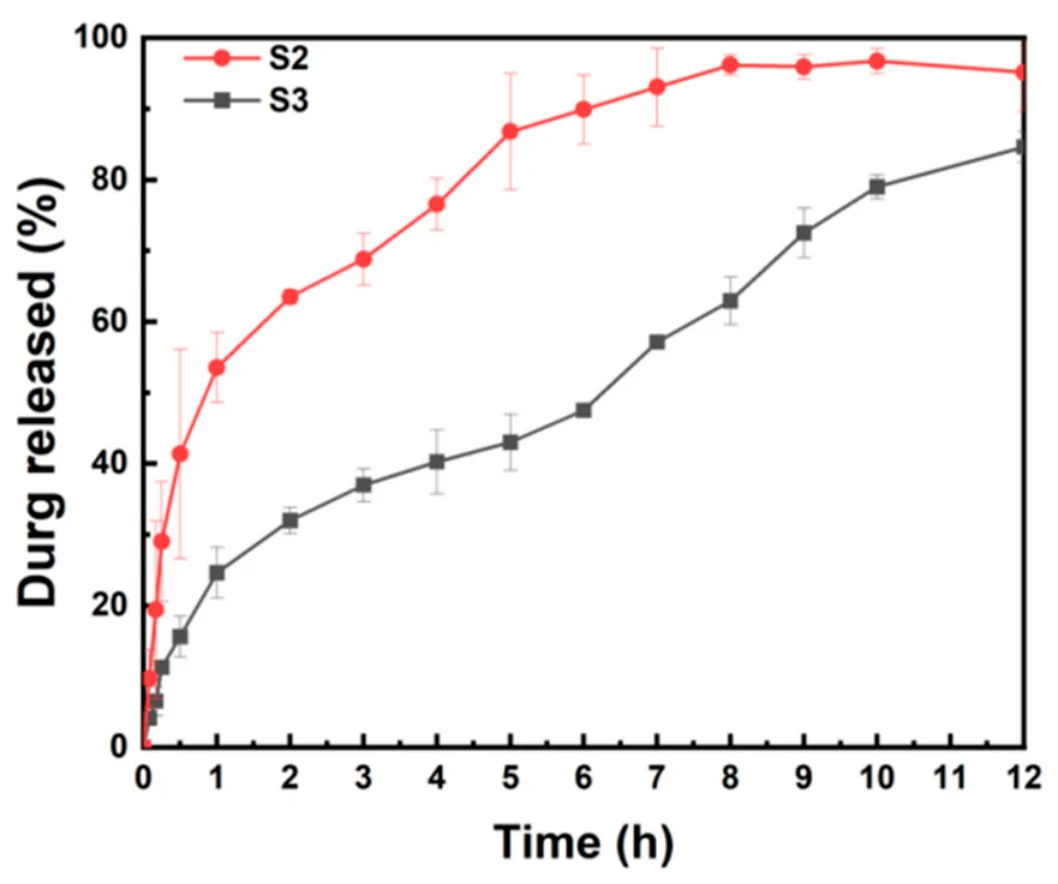
In vitro drug release profiles of S2 (loaded with only DP) and S3 (loaded with only SH) drug-loaded nanofibers.

This study successfully developed a novel tri-chamber structure for supporting the desired process–structure–performance relationship, in which the crescent-shaped PVP layer and the circular PLA core–sheath layer were integrated through a single step and a straightforward manner, enabling the differential loading and precise controlled release of two drugs. The drug release profiles are shown in Figure 9. In nanofibers S2, DP was loaded onto the hydrophilic crescent-shaped PVP layer. Its in vitro release profile exhibited typical biphasic release characteristics: rapid burst release from 0 to 1 h, with a cumulative release rate of approximately 53% at 1 h; followed by sustained slow release from 1 to 10 h, with a cumulative drug release rate approaching 100% within 10 h. This ensured both rapid drug onset and sustained-release properties. The strange sustained release of DP should be attributed to the insolubility of DP molecules, which clung on the hydrophobic surfaces of PLA side core–sheath composites.

In nanofibers S3, SH was loaded into a hydrophobic PLA core layer, significantly slowing the drug release rate and exhibiting a steady, gradual sustained-release profile. The cumulative release rate at 12 h was approximately 85%, with no noticeable burst release, meeting the design requirements for a long-acting controlled-release formulation. The release behavior of the two drugs closely aligns with their structural design: the rapid swelling of the PVP layer provides a rapid-release pathway for DP, while the dense barrier of the PLA core–sheath layer effectively delays the diffusion of SH, achieving a synergistic “rapid-release + sustained-release” controlled-release effect. The experimental results fully demonstrate that nanofibrous membranes with a tri-chamber structure integrated from a core–sheath structure and Janus fiber can achieve differentiated release of drugs with distinct pharmacological effects through precise control of drug loading positions. This provides a safe and highly efficient new carrier design for oral mucosal delivery systems—such as those for treating motion sickness—that require “rapid onset of action and long-lasting maintenance.” It should be noted that the in vitro dissolution profiles were calculated based on the theoretical drug loading content as the Q_0_ value. However, the Q_0_ value can also be the measured encapsulated content or the finally released drug content. All these methods have their own uncertainties, but their results should be similarly useful for evaluating drug release profiles, as the in vitro dissolution test itself is a rough evaluation method. The gold standard for evaluating controlled drug release remains the therapeutic effect after in vivo release [63,64,65,66,67].

Traditionally, PVP-based medicated polymeric composites often release the loaded drug in a rapid manner owing to its good solubility [68,69], whereas PLA-based polymeric products always release the loaded drug through the typical Fickian diffusion mechanism due to its insolubility and low swellability [70,71]. Therefore, two classical models were selected to systematically elucidate the release behavior and control mechanisms of the loaded drugs in the tri-chamber hierarchical nanofibers, i.e., zero-order and Ritger–Peppas—to fit and analyze the in vitro release data for S2 and S3. The zero-order kinetic model is characterized by a constant drug release rate that is independent of the remaining drug concentration; it represents the target release behavior for ideal sustained-release formulations. The Ritger–Peppas model is the gold standard for distinguishing drug release mechanisms; the release type can be accurately determined using the release index n: n < 0.45 indicates Fickian diffusion (solely diffusion-driven) [72], while 0.45 < n < 0.89 indicates non-Fickian diffusion (a synergistic effect of drug diffusion and matrix erosion), and n > 0.89 suggests an erosion mechanism.

**Figure 10:**
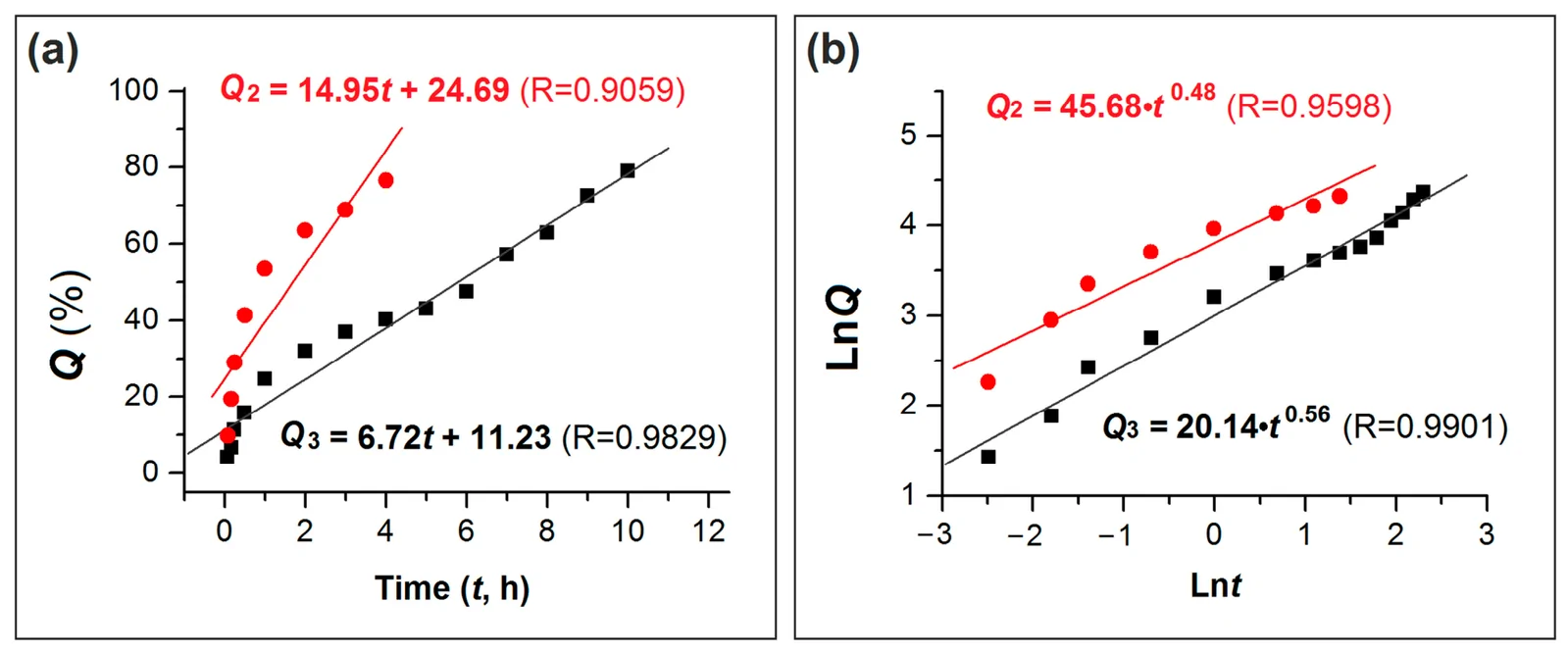
Drug release behaviors and mechanisms of S2 and S3 drug-loaded nanofibers: (**a**) zero-order kinetics model; and (**b**) Ritger–Peppas kinetics model.

The fitting results are included in Figure 10. As indicated in Figure 10a, SH from the nanofibers S3 fit well into the zero-order release behaviors with a correlation coefficient of R = 0.9829 for its equation of Q_3_ = 6.72*t* + 11.23, meeting the targeted drug release design. However, DP from the nanofibers S2 showed a worse fitting result, with a correction coefficient of R = 0.9059 for its equation of Q_2_ = 14.85*t* + 24.69. The release behaviors of DP from S2 were mainly controlled by the PVP matrices. However, the presence of the other hydrophobic PLA sides and also the hydrophobicity of DP would exert profound influences on the release behaviors of DP molecules, making the apparent release profiles very complicated. The fitting results according to the Ritger–Peppas model indicated an equation of Q_2_ = 45.68*t*^0.48^ (R = 0.9598) (Figure 10b) for the release of DP from nanofibers S2. The value of n = 0.48 > 0.45 suggested a combined mechanism of erosion and Fickian diffusion. However, drug molecules released from pure PVP matrices are always manipulated by polymeric erosion or dissolution mechanisms. Here, the deviations should be attributed to the other PLA sides and DP molecules could aggregate on the surface of PLA surfaces. The fitted Ritger–Peppas equation for SH from the nanofibers S3 is Q_3_ = 20.14 *t*^0.56^ (R = 0.9901) (Figure 10b). The value of n = 0.56 > 0.45 still suggested a combined mechanism of Fickian diffusion and erosion. However, PLA is not soluble in water and has low swelling performance. The deviations of release mechanisms from the traditional Fickian diffusion mechanism should be attributed to the following factors: (1) the influence of the soluble side of PVP on the pseudo-equilibrium of PLA; (2) and the general requirements for the applicability of the Ritger–Peppas equation, which include a drug uniformly distributed in the matrix, a dosage form with a well-defined/regular geometry, and at the early stage. Nonetheless, the apparent release behaviors of both drugs align closely with the design of the tri-chamber side-by-side-coaxial structure, achieving the desired synergistic complex controlled release of “immediate-release + sustained-release” and providing a novel carrier strategy for oral mucosal administration in the treatment of motion sickness.

By eliminating the initial burst release effect, zero-order release profiles can be achieved from electrospun core–sheath nanofibers containing a water-insoluble polymeric blank sheath coating [73,74]. Moreover, the thickness of the blank sheath can be exploited as a key parameter to tailor the rate of sustained drug release [75]. Here, an insoluble core–sheath nanostructure with a blank sheath coating was laterally attached to crescent-shaped water-soluble polymer nanocomposites (the PVP-DP sides). The pulsatile release of DP from the PVP matrices and the sustained release of SH from the PLA core–sheath structures combined to produce the desired complex asynchronous release profile. Such complex release kinetics results from the rational selection of polymeric matrices, the multi-chamber nanostructure, and the appropriate spatial distribution of the loaded active ingredients. Building on this proof-of-concept demonstration, the composition of the components, the thickness of the sheath sections, and the spatial distribution of the loaded drugs can be further exploited to manipulate the release behaviors of the drug molecules.

Compared to the popular core–sheath dual-drug-loaded oral films prepared by coaxial electrospinning, the tri-chamber nanofibers innovatively designed in this study further validate the advantages of multi-chamber structures for long-acting controlled release, more precisely meeting the clinical requirements for anti-motion sickness drugs—namely, “rapid onset of action and long-lasting maintenance”—and providing an optimal structural design for dual-phase controlled-release oral films.

### 3.7. Cytotoxicity and Live-Dead Staining Assays

**Figure 11:**
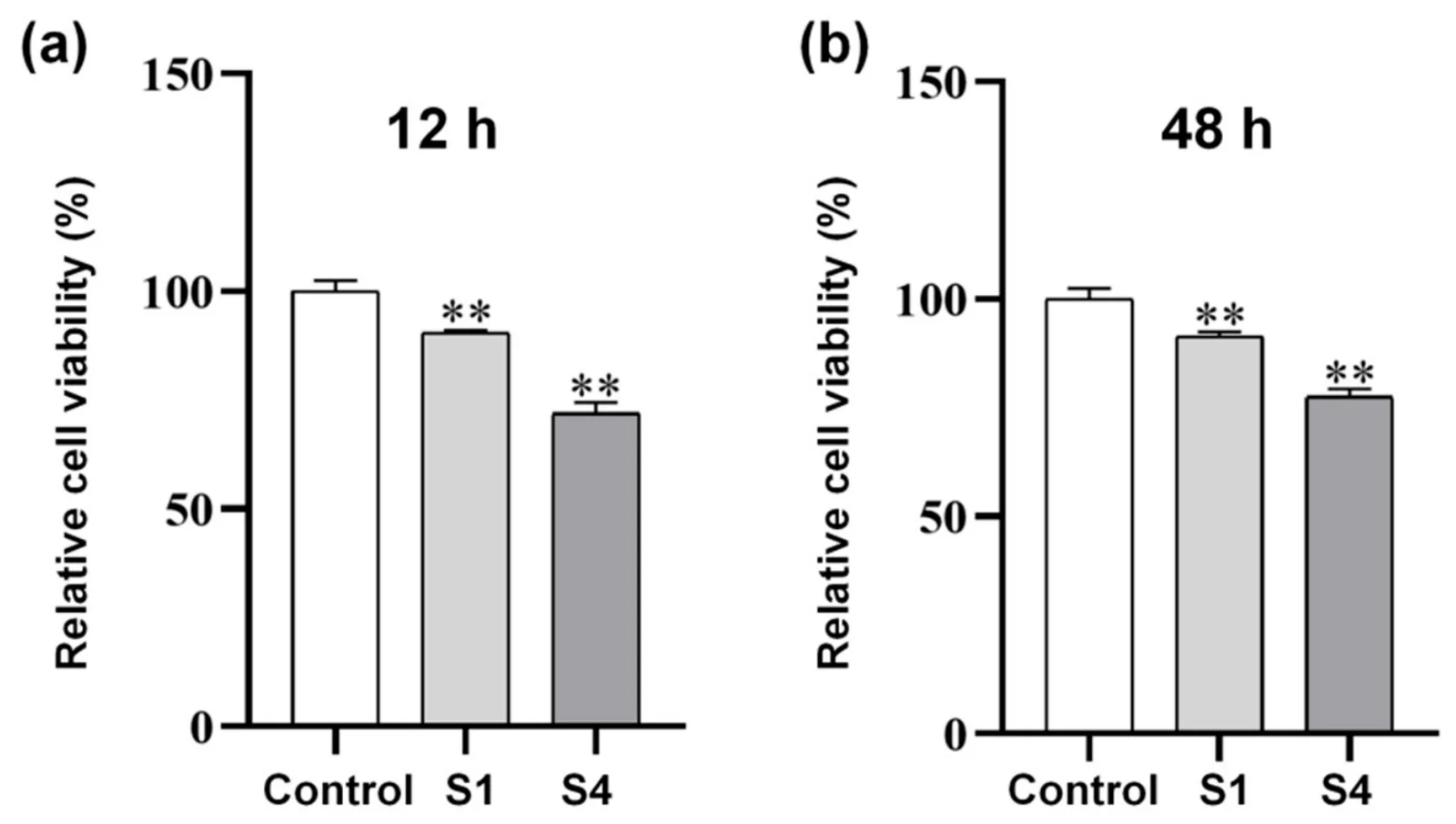
Relative cell viability of L929 cells after co-culture with samples: (**a**) 12 h of co-culture; (**b**) 48 h of co-culture. Data are normalized to the control group (Control). ** indicates a highly significant difference compared to the control group (*p* < 0.01).

Figure 11 presents the results of a cytotoxicity assay using the CCK-8 method to analyze cell viability in the drug-free nanofiber group (S1) and the dual-drug-loaded nanofiber group (S4). The relative cell viability of L929 fibroblasts co-cultured with the blank control, S1, and S4 for 12 h and 48 h was used to systematically evaluate the in vitro biocompatibility of the three-fluid coaxial nanofiber oral membrane. The results show that, compared with the control group, cell viability in the S1 and S4 nanofibers decreased significantly at both 12 h and 48 h (*p* < 0.01). Specifically, at 12 h, cell viability in the S1 and S4 nanofibers was approximately 90% and 75%, respectively; at 48 h, it was approximately 90% and 80%, respectively. Although the pharmacological activity of the drugs exerted a certain degree of reversible inhibition on cell proliferation, cell viability in the S4 nanofibers remained above 80% at 48 h, meeting the safety standards for biomedical materials [76]. This demonstrates that the dual-drug-loaded nanofiber membrane exhibits acceptable in vitro biosafety and does not induce strong cytotoxicity, providing a solid biological basis for its potential clinical application as an oral mucosal drug delivery system for motion sickness.

**Figure 12:**
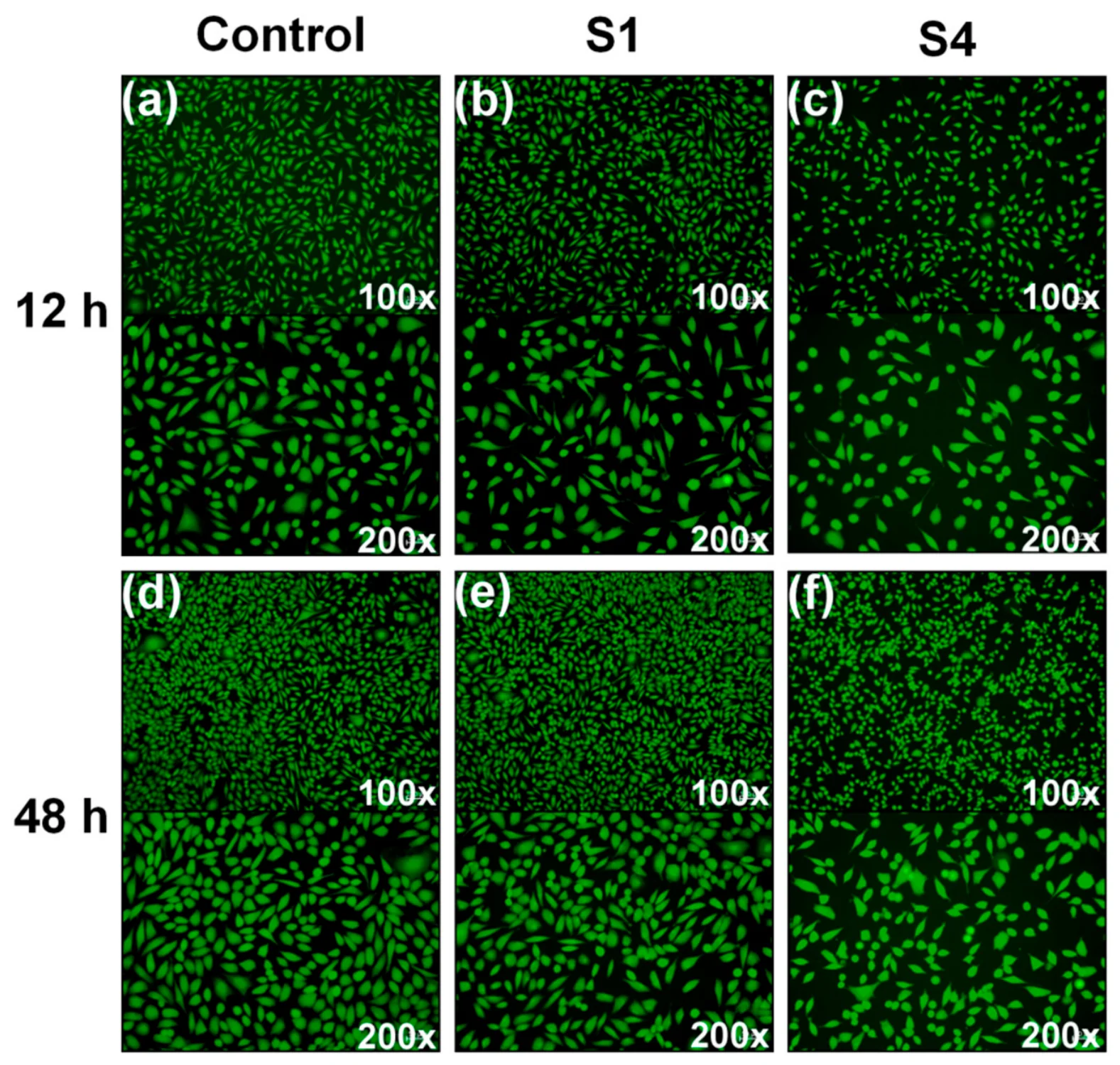
Fluorescence staining results of live L929 cells after 12 h and 48 h of co-culture with samples: 12 h co-culture (**a**) control group, (**b**) drug-free group S1, (**c**) dual-drug co-loaded group S4; 48 h co-culture (**d**) control group, (**e**) drug-free group S1, (**f**) dual-drug co-loaded group S4.

Figure 12 shows the results of fluorescent staining of live L929 fibroblasts after 12 h and 48 h of co-culture with the blank control (Control), the drug-free nanofiber membrane S1, and the dual-drug-loaded nanofiber membrane S4. The images, taken at 100× and 200× magnification, visually illustrate the cells’ viability, morphology, and proliferation status. The results show that at both the 12 h and 48 h time points, cells in the control, S1, and S4 groups exhibited distinct green fluorescence. The cells maintained intact morphology with a typical spindle-shaped structure characteristic of fibroblasts; they displayed high density and uniform distribution, with no obvious signs of cell shrinkage, fragmentation, or death. There was virtually no difference in cell density or morphology between the S1 and control groups. Although the S4 group exhibited a slight decrease in cell density due to the pharmacological activity of the drugs, it still maintained good cell viability and normal cell morphology, with limited apparent cytotoxic damage. These results corroborate the quantitative CCK-8 findings in Figure 11, further confirming at the qualitative level that both the drug-free S1 and the dual-drug-loaded S4 nanofiber membranes exhibit excellent in vitro biocompatibility with slight cytotoxicity. The dual-drug loading did not cause irreversible damage to L929 cells, fully meeting the safety requirements for biomedical materials used in oral mucosal patches and providing direct biological support for the clinical application of this three-fluid coaxial nanofiber membrane.

Although the present investigation successfully moved forward from the coaxial electrospinning and core–sheath nanofibers to trifluid multi-channel electrospinning and hierarchical nanofibers, determining how to produce on a large scale for potential commercial products presents one of the most difficult challenges. Besides these concerns, other potential future investigations include, but are not limited to, the following topics: (1) the animal and clinic investigations of the developed mucous dosage forms; (2) other kinds of hierarchical nanostructures integrated from the core–sheath and Janus bi-chamber structures, such as core–sheath structures with an inner Janus core, or core–sheath structures with a core to support two Janus sheaths, and even quad-chambers consisting of two core–sheath structures in a Janus manner; (3) further tailoring the components and compositions of the three chambers, such as co-loaded flavoring agents at the PVP sides, or transmembrane enhancers [77,78,79]; and (4) green electrospinning processes with bio-polymers and organic solvent-free working processes [80,81,82,83]. As for the large-scale nanofabrication issue, Yu et al. recently developed a new electrospinning technique for batch production of nanofibers, which is an integrated method based on traditional needle-based electrospinning and free surface electrospinning [84,85]. Based on the combination of the idea verified here and the new electrospinning method, large-scale production of the Janus nanofibers reported in this article is under investigation. It should be noted that the asynchronous co-delivery of DP and SH from Janus sample S4 was inferred from the combined results of S2 and S3, which shared a similar Janus structure to that of S4 but were each loaded with a single drug on one side. This arrangement was necessitated by the closely spaced absorption maxima of DP and SH, which rendered UV–Vis measurements of co-presence solutions invalid. Therefore, by referring to the literature [86], a similar method based on UV–Vis absorbance using single-drug-loaded multi-chamber structures was developed for quantitative evaluation. In the future, with high-performance liquid chromatography as the detection tool and under suitable measurement conditions, direct demonstration can be achieved.

## 4. Conclusions

In this study, a custom-built modified trifluid electrospinning device incorporating a coaxial structure was successfully developed to prepare a dual-drug-loaded nanofiber membrane with a segmented structure consisting of a PVP crescent chamber, a PLA sheath chamber, and a PLA core chamber. SEM and TEM results indicate that the fiber morphology is continuous and smooth, with clear interfaces between the three chambers. DP and SH are localized on the crescent-shaped side and the circular core layer, respectively, without interfering with each other, and the introduction of drugs optimizes fiber diameter uniformity. FTIR and XRD analyses confirm that the two drugs exist in a physically dispersed and amorphous state within their respective regions, without undergoing chemical reactions with the polymer or disrupting the matrix structure. Mechanical and wettability tests showed that the membrane exhibits balanced flexibility and rigidity; the incorporation of DP significantly enhanced its hydrophilicity and wetting rate, thereby facilitating its adhesion to the oral mucosa. Using two types of hierarchically structured nanofibers S2 and S3, each loaded with a single drug to facilitate measurement, the in vitro drug release studies demonstrated a synergistic controlled-release profile with rapid release of DP and sustained release of SH, with the sustained-release performance outperforming that of core–sheath fibers. The former was primarily driven by Fickian diffusion, while the latter resulted from a combination of diffusion and matrix dissolution. CCK-8 and live/dead staining results indicated that the drug-loaded fiber membrane exhibited low cytotoxicity toward L929 cells and acceptable biocompatibility. This trifluid electrospinning technique enables precise spatial partitioning and differentiated controlled release of the two drugs. The resulting nanofiber membranes are the potential candidates for drug delivery to the oral mucosa in terms of structure, physicochemical properties, drug release behavior, and biosafety, providing a reliable experimental basis and carrier foundation for a synergistic fast-release/sustained-release drug delivery system for motion sickness.

## 5. Patents

Yu, D.G.; Yang, J.; Zhang, L.; Zhang, Y. One Side-by-Side Spinneret with A Coaxial Side, Its Spinning Apparatus, and the Implementation Method. Chinese Invention Patent, No. ZL201611004513.5, Authorized: 15 February 2019.

## Figures and Tables

**Table 1:** Experimental parameters for the preparations of nanofibers ^a^.

Fibers No.	Core Layer		Sheath Layer	Crescent Layer		Flow Rate Ratio (mL/h)
	Polymer	Medicine	Polymer	Polymer	Medicine	
S1	PLA	\	PLA	PVP K90	\	1:2:1.5
S2	PLA	\	PLA	PVP K90	DP	1:2:1.5
S3	PLA	SH	PLA	PVP K90	\	1:2:1.5
S4	PLA	SH	PLA	PVP K90	DP	1:2:1.5

^a^ The applied voltage and the nanofibers deposition distance were fixed at 12 kV and 20 cm, respectively. The solvent for PLA solution was TFEA, and the PVP K90 solution was prepared using a solvent mixture of TFEA and deionized water with a volume ratio of 9.5:0.5.

## Data Availability

The data that support the findings of this study are available from the corresponding author upon reasonable request.

## References

[1] Qu H., Wei S., Guo Z. (2013). Coaxial Electrospun Nanostructures and Their Applications. J. Mater. Chem. A.

[2] Kamboj M., Kaur J., Rathi V., Poundarik A., Preet S., Das B. (2026). A Multiphasic Core–shell Flurbiprofen and Ciprofloxacin-loaded Nanofibrous Dressing Cures Infected Burns via Dual Control of Infection and Inflammatory Response. Biomater. Sci..

[3] Zhou J., Xue F., Xu L., Liao F., Yu D.-G., Ge R. (2026). Comparative Study of Bio-based and Synthetic Phase Change Materials Functionalized Coaxial Fibers for Photothermal-controlled Drug Delivery. Biomater. Adv..

[4] Yao Z.-C., Wang J.-C., Ahmad Z., Li J.-S., Chang M.-W. (2019). Fabrication of Patterned Three-dimensional Micron Scaled Core-sheath Architectures for Drug Patches. Mater. Sci. Eng. C.

[5] Ye P., Yusufu R., Guan Z., Chen T., Li S., Feng Y., Zeng X., Lu J., Luo M., Wei F. (2024). Multifunctional Bioactivity Electrospinning Nanofibers Encapsulating Emodin Provide a Potential Postoperative Management Strategy for Skin Cancer. Pharmaceutics.

[6] Dong R., Gong W., Guo Q., Liu H., Yu D.-G. (2024). Synergistic Effects of Radical Distributions of Soluble and Insoluble Polymers within Electrospun Nanofibers for an Extending Release of Ferulic Acid. Polymers.

[7] Sun L., Zhao Y., Caomeng X., Yu D.-G., Liu P. (2026). Core–Sheath Nanofibers from a Modified Coaxial Electrospinning for Transdermal Delivery of Finasteride. ChemistryOpen.

[8] Si Y., Shi S., Zhi C., Lei L., Hu J., Yang L. (2025). Janus Electrospun Membranes. Adv. Mater..

[9] Wang Q., Chen T., Dou H., Yu D.-G. (2026). Electrospun Integrated Janus Nanofibers for Asynchronous Delivery of Finasteride and Tibetan Medicine. Pharmaceutics.

[10] Gupta P., Wilkes G.L. (2003). Some Investigations on the Fiber Formation by Utilizing a Side-by-side Bicomponent Electrospinning Approach. Polymer.

[11] Lv D., Liu H., Li D., Wang T., Yang Y., Yu W., Yin D., Bo X., Dong X. (2026). Mo2C and WP Embedded in P-Doped Janus Carbon Fibers as a Highly Active Electrocatalyst for Acidic and Alkaline Hydrogen Evolution Reactions. ACS Appl. Mater. Interfaces.

[12] Yang X., Qi H., Jiang S., Dong X., Duan G. (2022). Di-aeolotropic Electric Conductive Janus Nanobelts with Up-/down-converting Fluorescence and Magnetism by Sequential Coaxial and Side-by-side Electrospinning. Compos. Commun..

[13] Walther A., Müller A.H.E. (2013). Janus Particles: Synthesis, Self-assembly, Physical Properties, and Applications. Chem. Rev..

[14] Chen S., Yun C., Wu X., Ding X. (2026). One-step Eccentric Side-by-side Electrospraying of Janus Particle Coatings on Cotton in an Ethanol–water System for Multifunctional Finishing. Prog. Org. Coat..

[15] Wang B., Yang M., Yao R., Zhao H., Yu D., Du L., Zou S., Zhu Y. (2026). Waterproof Fabric with Copper Ion-Loaded Multicompartmental Nanoparticle Coatings for Jellyfish Repellency. Pharmaceutics.

[16] Ma Q., Yu W., Dong X., Wang J., Liu G., Xu J. (2012). Electrospinning Preparation and Properties of Fe_3_O_4_/Eu(BA)_3_phen/PVP Magnetic-photoluminescent Bifunctional Composite Nanofibers. J. Nanopart. Res..

[17] Lu H., Wang Q., Li G., Qiu Y., Wei Q. (2017). Electrospun Water-stable Zein/ethyl Cellulose Composite Nanofiber and Its Drug Release Properties. Mater. Sci. Eng. C.

[18] Li H., Limaye A., Osorno L., Alheib O., Wang Z., Ievlev A.V., Domingo N., Arinzeh T., Foston M. (2025). Spatial Distribution and Clustering of Glycosaminoglycans in Electrospun Gelatin-Based Scaffolds. ACS Omega.

[19] Wang J.-C., Zheng H., Chang M.-W., Ahmad Z., Li J.-S. (2017). Preparation of Active 3D Film Patches via Aligned Fiber Electrohydrodynamic (EHD) Printing. Sci. Rep..

[20] Ruchika, Yadav S.K., Saneja A. (2025). Electrospun Proliposomes and the Quest to Overcome Liposome Instability. Drug Discov. Today.

[21] Chrysanthou C., Manikion K., Voniatis C. (2026). The Potential of Electrospun Meshes in Postoperative Pain Management. Pharmaceutics.

[22] Yao S., Wang M., Yang L., Wang H., Wang A.L., Zhang Y., Cui W., Wang L. (2026). The Coupling of Gradient Mechano-Electrical Microenvironments Promotes Interface Regeneration via Ca^2+^ Signaling Activation. Adv. Funct. Mater..

[23] Chaudhari D.R., Ramani V.D., Shah D.P., Vaishnav D., Kapoor D.U. (2024). A Synoptic Examination of Commercially Viable Nanofiber-based Products. J. Drug Deliv. Sci. Technol..

[24] Omer S., Forgách L., Zelkó R., Sebe I. (2021). Scale-up of Electrospinning: Market Overview of Products and Devices for Pharmaceutical and Biomedical Purposes. Pharmaceutics.

[25] Tabakoglu S., Kołbuk D., Sajkiewicz P. (2023). Multifluid Electrospinning for Multi-drug Delivery Systems: Pros and Cons, Challenges, and Future Directions. Biomater. Sci..

[26] Zhang X., Chi C., Chen J., Zhang X., Gong M., Wang X., Yan J., Shi R., Zhang L., Xue J. (2021). Electrospun Quad-axial Nanofibers for Controlled and Sustained Drug Delivery. Mater. Des..

[27] Xing J., Feng J., Wang Q., Yu D.-G., Annie Bligh S.-W. (2026). Recent Advances in Electrospun Nanofiber-based Implantable Drug Delivery Systems for Breast Cancer Therapy. RSC Pharm..

[28] Tabakoglu S., Kołbuk D., Sajkiewicz P. (2025). Triaxial Electrospun Nanofiber Membranes for Prolonged Curcumin Release in Dental Applications: Drug Release and Biological Properties. Molecules.

[29] Cho Y., Baek J.W., Sagong M., Ahn S., Nam J.S., Kim I.-D. (2025). Electrospinning and Nanofiber Technology: Fundamentals, Innovations, and Applications. Adv. Mater..

[30] Oleiwi A.H., Dalfi H.K. (2026). A Comprehensive Review on Electrospinning: Mechanisms, Functional Modifications and the Importance of Nanofiber Systems. Int. J. Nanosci..

[31] Yang X., Wang Q., Zhu Z., Lu Y., Liu H., Yu D.-G., Bligh S.-W.A. (2025). A Modified Triaxial Electrospinning for a High Drug Encapsulation Efficiency of Curcumin in Ethylcellulose. Pharmaceutics.

[32] Silva P.M., Torres-Giner S., Vicente A.A., Cerqueira M.A. (2022). Management of Operational Parameters and Novel Spinneret Configurations for the Electrohydrodynamic Processing of Functional Polymers. Macromol. Mater. Eng..

[33] Keirouz A., Wang Z., Reddy V.S., Nagy Z.K., Vass P., Buzgo M., Ramakrishna S., Radacsi N. (2023). The History of Electrospinning: Past, Present, and Future Developments. Adv. Mater. Technol..

[34] Jiang H., Wang L., Zhu K. (2014). Coaxial Electrospinning for Encapsulation and Controlled Release of Fragile Water-soluble Bioactive Agents. J. Control. Release.

[35] Wang S., Chen W., Wang L., Yao J., Zhu G., Guo B., Militky J., Venkataraman M., Zhang M. (2022). Multifunctional Nanofiber Membrane with Anti-ultraviolet and Thermal Regulation Fabricated by Coaxial Electrospinning. J. Ind. Eng. Chem..

[36] Chen Y., Gong W., Zhang Z., Zhou J., Yu D.-G., Yi T. (2024). Reverse Gradient Distributions of Drug and Polymer Molecules within Electrospun Core–Shell Nanofibers for Sustained Release. Int. J. Mol. Sci..

[37] Wang P., Liu Y., He W., Chen Y., Zhou J., Yu D.-G. (2025). Coaxial Electrospun ZIF-8@PAN Nanofiber Membranes for Tetracycline and Doxycycline Adsorption in Wastewater. Adv. Mater. Interfaces.

[38] Yoon J., Yang H.-S., Lee B.-S., Yu W.-R. (2018). Recent Progress in Coaxial Electrospinning: New Parameters, Various Structures, and Wide Applications. Adv. Mater..

[39] Xiao Y., Xie F., Luo H., Tang R., Hou J. (2022). Electrospinning SA@PVDF-HFP Core–Shell Nanofibers Based on a Visual Light Transmission Response to Alcohol for Intelligent Packaging. ACS Appl. Mater. Interfaces.

[40] Yu D.G., Wang X., Zou H., Xu Y., Yang C. (2017). A Special Side-by-Side Spinneret Surrounded by a Teflon Tube and Its Application. Chinese Authorized Patent.

[41] Yu D.G., Yang J., Zhang L., Zhang Y. (2019). One Side-by-Side Spinneret with a Coaxial Side, Its Spinning Apparatus, and the Implementation Method. Chinese Invention Patent.

[42] Wang C., Wang W., Qi H., Dai Y., Jiang S., Ding B., Wang X., Li C., Zeng J., Wu T. (2025). Electrospinning and Electrospun nanofibers: From Academic Research to Industrial Production. Prog. Mater. Sci..

[43] Hu Y., Zhang F., Zhou J., Yang Y., Ding D., Li M., Wang C., Wang B., Yu J., Jiang F. (2025). Electrospun Tri-Layer Core-Sheath Nanofibrous Coating for Sequential Treatment of Postoperative Complications in Orthopedic Implants. Small.

[44] Zheng Y., Meng N., Xin B. (2018). Effects of Jet Path on Electrospun Polystyrene Fibers. Polymers.

[45] Xu C., He W., Yang W., Deng W., Xia H. (2022). Controlling Instabilities of Electrified Liquid Jets via Orthogonal Perturbations. Phys. Rev. Fluids.

[46] Porwal M., Chandra P., Sridhar S.B., Shareef J., Wadhwa T., Malviya R. (2026). Physicochemical Science of Electrospun Nanofibers as Drug Delivery Systems for Improved Clinical Outcomes. Polym. Adv. Technol..

[47] Özkan A., Özkan V. (2026). Process–Structure–Property Engineering of Bacterial Cellulose Nanofibers via Sustainable Biosynthesis and Green Electrospinning. Fibers Polym..

[48] Gupta D., Soni S. (2026). Electrospun Nanofibers in Wound Management: A Comprehensive Review of Fabrication, Functionality, and Future Directions. Polym. Adv. Technol..

[49] Zhao Y., Cao X., Jiang L. (2007). Bio-Mimic Multichannel Microtubes by A Facile Method. J. Am. Chem. Soc..

[50] He W., Lv H., Feng J., Yi W., Wang H., Yu D.G., Yi T. (2026). Electrospun Janus Nanofibrous Mats as Multifunctional ish Preservation Gaskets. Macromol. Mater. Eng..

[51] Squires T.M., Quake S.R. (2005). Microfluidics: Fluid physics at the nanoliter scale. Rev. Mod. Phys..

[52] Yu D.-G., Li J.-J., Williams G.R., Zhao M. (2018). Electrospun Amorphous Solid Dispersions of Poorly Water-soluble Drugs: A Review. J. Control. Release.

[53] Lu Y., Dong R., Wang B., Liu H., Yu D.-G., Zhu Y. (2026). Janus Films Fabricated Using Modified Electrospinning for Converting Dosage Form of Watermelon Frost. ACS Omega.

[54] Choudhary S. (2018). Structural, Morphological, Thermal, Dielectric, and Electrical Properties of Alumina Nanoparticles Filled PVA–PVP Blend Matrix-Based Polymer Nanocomposites. Polym. Compos..

[55] Holder C.F., Schaak R.E. (2019). Tutorial on Powder X-ray Diffraction for Characterizing Nanoscale Materials. ACS Nano.

[56] Khan H., Yerramilli A.S., D’Oliveira A., Alford T.L., Boffito D.C., Patience G.S. (2020). Experimental Methods in Chemical Engineering: X-Ray Diffraction Spectroscopy—XRD. Can. J. Chem. Eng..

[57] Hanchar J.M., Nagy K.L., Fenter P., Finch R.J., Beno D.J., Sturchio N.C. (2020). Quantification of Mminor Phases in Growth Kinetics Experiments with Powder X-Ray Diffraction. Am. Mineral..

[58] Lai K.L., Fan Y., Han H., Li Q., Zhang S., Li H.Y., Chow S.F., Lam T.N., Lee W.Y.T. (2018). Orally-Dissolving Film for Sublingual and Buccal Delivery of Ropinirole. Colloids Surf. B Biointerfaces.

[59] Cheng H., Wang Y., Hong Y., Wu F., Shen L., Lin X. (2025). Characteristics, Preparation and Applicability in Oral Delivery Systems of Cellulose Ether–Based Buccal Films. Drug Deliv..

[60] Zahra F.T., Zhang Y., Ajayi A.O., Quick Q., Mu R. (2024). Optimization of Electrospinning Parameters for Lower Molecular Weight Polymers: A Case Study on Polyvinylpyrrolidone. Polymers.

[61] Yang N., Williams G.R., Jalil B., Mykhailenko O. (2026). Electrospun Composite Lavandula Angustifolia Mill. Extract/PVP Nanofibers. Macromol. Mater. Eng..

[62] Kang S., Hou S., Chen X., Yu D.-G., Wang L., Li X., Williams G.R. (2020). Energy-Saving Electrospinning with a Concentric Teflon-Core Rod Spinneret to Create Medicated Nanofibers. Polymers.

[63] Wang L., Liu W., Jiang Q., Wang X., Xu D., Fang Y., Wang S., Tang J. (2026). Span Value as a Critical Quality Attribute for PLGA Microspheres: Controlling Burst Release and Enhancing Therapeutic Efficacy via Wet Sieving. Pharmaceutics.

[64] Xu H., Liu Y., Jin L., Chen X., Chen X., Wang Q., Tang Z. (2023). Preparation and Characterization of Ion-Sensitive Brimonidine Tartrate In Situ Gel for Ocular Delivery. Pharmaceuticals.

[65] Fredenberg S., Wahlgren M., Reslow M., Axelsson A. (2011). The Mechanisms of Drug Release in Poly(Lactic-Co-Glycolic Acid)-Based Drug Delivery Systems—A Review. Int. J. Pharm..

[66] D’Souza S.S., DeLuca P.P. (2006). Methods to Assess In Vitro Drug Release from Injectable Polymeric Particulate Systems. Pharm. Res..

[67] Shen J., Burgess D.J. (2015). In Vitro–In Vivo Correlation for Complex Non-Oral Drug Products: Where Do We Stand?. J. Control. Release.

[68] Luo Y., Hong Y., Shen L., Wu F., Lin X. (2021). Multifunctional Role of Polyvinylpyrrolidone in Pharmaceutical Formulations. AAPS PharmSciTech.

[69] Franco P., De Marco I. (2020). The Use of Poly(N-Vinyl Pyrrolidone) in the Delivery of Drugs: A Review. Polymers.

[70] Miyajima M., Koshika A., Okada J., Ikeda M. (1999). Mechanism of Drug Release from Poly(L-Lactic Acid) Matrix Containing Acidic or Neutral Drugs. J. Control. Release.

[71] Thamvasupong P., Viravaidya-Pasuwat K. (2022). Controlled Release Mechanism of Vancomycin from Double-Layer Poly-L-Lactic Acid-Coated Implants for Prevention of Bacterial Infection. Polymers.

[72] Peppas N.A. (1985). Analysis of Fickian and Non-Fickian Drug Release from Polymers. Pharm. Acta Helv..

[73] Li Z., Kang H., Che N., Liu Z., Li P., Li W., Zhang C., Cao C., Liu R., Huang Y. (2014). Controlled release of liposome-encapsulated Naproxen from core-sheath electrospun nanofibers. Carbohydr. Polym..

[74] Pant B., Park M., Park S.-J. (2019). Drug Delivery Applications of Core-Sheath Nanofibers Prepared by Coaxial Electrospinning: A Review. Pharmaceutics.

[75] Wang C., Yan K.-W., Lin Y.-D., Hsieh P.C.H. (2010). Biodegradable Core/Shell Fibers by Coaxial Electrospinning: Processing, Fiber Characterization, and Its Application in Sustained Drug Release. Macromolecules.

[76] Guadix J.A., López-Beas J., Clares B., Soriano-Ruiz J.L., Zugaza J.L., Gálvez-Martín P. (2019). Principal Criteria for Evaluating the Quality, Safety and Efficacy of hMSC-Based Products in Clinical Practice: Current Approaches and Challenges. Pharmaceutics.

[77] Yu X., Xu Y., Wang B., Yu D.-G., Zhang M., Zhu Y. (2026). Batch Sequential Production of Medicated Janus Fibrous Films of Polyvinylpyrrolidone/Shellac for Treating Pharyngitis. RSC Adv..

[78] Abdelhakim H.E., Coupe A., Tuleu C., Edirisinghe M., Craig D.Q.M. (2021). Utilising Co-Axial Electrospinning as a Taste-Masking Technology for Paediatric Drug Delivery. Pharmaceutics.

[79] Shokoya M.M., Szabó E., Nagy Z.K., Sebe I., Zelkó R. (2026). Therapeutic Nanofibrous Scaffolds in Precision Drug Delivery: Formulation Design, Challenges and Regulatory Frameworks. Expert Opin. Drug Deliv..

[80] Sun Y., Zhou J., Zhang Z., Yu D.-G., Bligh S.W.A. (2024). Integrated Janus Nanofibers Enabled by a Co-shell Solvent for Enhancing Icariin Delivery Efficiency. Int. J. Pharm..

[81] Berdimurodov E., Dagdag O., Berdimuradov K., Wan Nik W.M.N., Eliboev I., Ashirov M., Niyozkulov S., Demir M., Yodgorov C., Aliev N. (2023). Green Electrospun Nanofibers for Biomedicine and Biotechnology. Technologies.

[82] Sabeghi Y., Pawase P.A. (2026). Electrospun Zein Nanofibers for Food-Contact Applications: Bioactive Delivery, Active, and Intelligent Systems. J. Food Process Eng..

[83] Morais M.S., Chagas P.A.M., da Mata G.C., Silva G.R., De Martinis E.C.P., Pinto G.H.A., Galvão G.F., Aguiar M.L., Oliveira W.P. (2026). Electrospun Polymeric Nanofibers Incorporating Brazilian Red Propolis Extract for Wound Dressing Applications. Pharmaceutics.

[84] Dong R., Ru Y., Zhang X., Zhu J., Wen H., He D., Yu D.-G. (2026). Scalable and Systematical Conversions of Domestic Wastes to Core–Sheath Nanofibers for Photocatalytic Degradation of Antibiotics. ACS Omega.

[85] Chen T., Guo Q., Yin H., Wang M., Liu H., Yu D.-G. (2026). Batch Productions of Electrospun Janus Nanofibrous Films for Multifunctional Food-Packaging Applications. Foods.

[86] Zhao P., Zhou K., Xia Y., Qian C., Yu D., Xie Y., Liao Y. (2024). Electrospun Trilayer Eccentric Janus Nanofibers for a Combined Treatment of Periodontitis. Adv. Fiber Mater..

